# Volumetric Absorptive Microsampling of Blood for Untargeted Lipidomics

**DOI:** 10.3390/molecules26020262

**Published:** 2021-01-07

**Authors:** Camilla Marasca, Maria Encarnacion Blanco Arana, Michele Protti, Andrea Cavalli, Laura Mercolini, Andrea Armirotti

**Affiliations:** 1 Computational and Chemical Biology, Fondazione Istituto Italiano di Tecnologia, Via Morego 30, 16163 Genova, Italy; camilla.marasca@iit.it (C.M.); andrea.cavalli@iit.it (A.C.); 2 Research Group of Pharmaco-Toxicological Analysis (PTA Lab), Department of Pharmacy and Biotechnology (FaBiT), Alma Mater Studiorum-University of Bologna, Via Belmeloro 6, 40126 Bologna, Italy; michele.protti2@unibo.it (M.P.); laura.mercolini@unibo.it (L.M.); 3 Analytical Chemistry Lab, Fondazione Istituto Italiano di Tecnologia, Via Morego 30, 16163 Genova, Italy; maria.blanco@gmail.com

**Keywords:** lipidomics, microsampling, whole blood, VAMS, DBS

## Abstract

In the present, proof-of-concept paper, we explore the potential of one common solid support for blood microsampling (dried blood spot, DBS) and a device (volumetric absorptive microsampling, VAMS) developed for the untargeted lipidomic profiling of human whole blood, performed by high-resolution LC-MS/MS. Dried blood microsamples obtained by means of DBS and VAMS were extracted with different solvent compositions and compared with fluid blood to evaluate their efficiency in profiling the lipid chemical space in the most broad way. Although more effort is needed to better characterize this approach, our results indicate that VAMS is a viable option for untargeted studies and its use will bring all the corresponding known advantages in the field of lipidomics, such as haematocrit independence.

## 1. Introduction

Given the importance of lipids in all the biological processes of a living organism, lipidomics has become a very significant analytical tool in many fields of biology [1,2,3,4,5]. Mass spectrometry (MS) plays a major role in this topic, thanks to its flexibility, sensitivity, and, when exploited in untargeted mode, the possibility to efficiently profile hundreds of individual lipid species from tens of lipid families [6,7,8]. Reversed phase chromatography, when coupled to high-resolution MS and used with different elution systems, allows an efficient separation and identification of lipids often in a matter of minutes [9]. For many different reasons, mostly related to biomarker discovery, when untargeted lipidomics are envisaged for human studies, plasma has generally been the focus of attention [3,6,8,10,11]. Less has been done to explore the intact blood lipidome. Only a few studies on the subject can be found in literature, such as an analysis of eicosanoid in human whole blood [12]. This is mainly due to pre-analytical sample handling and processing drawbacks, and to the complexity of whole blood samples compared to plasma and serum derivatives [13]. To overcome the disadvantages associated to fluid whole blood analysis, several authors have exploited microsampling approaches, such as dried blood spot (DBS), widely used for neonatal screening for inborn errors of metabolism [14,15,16]. DBS involves a minimally invasive collection of blood (less than 50 µL), usually obtained from a heel or fingerprick, spotted onto filter paper cards, and followed by simplified storage and transportation procedures. These samples, simply dried at room temperature, usually do not require cryopreservation. After minimal training, DBS collection can be easily performed by non-qualified operators, thus representing a promising perspective for point-of-care or home-sampling. Due to its many advantages, DBS has been applied to many fields of bioanalysis, including pharmacokinetic studies, in order to improve animal welfare and reduce the number of animals needed [17]. Moreover, microsampling has also been exploited for proteomics to assess protein stability [18], for targeted metabolomics [15], for lipidomic studies based on tailored extraction procedures [19] and, more in general, for biomarker discovery [20,21]. DBS sampling has also been tested for shotgun lipidomics [22]. One miniaturised sampling technique is volumetric absorptive microsampling (VAMS), which holds all the advantages of DBS whilst implementing additional ones, mostly related to sampling volume accuracy and haematocrit (HCT)-dependent volumetric bias. VAMS devices are able to sample a fixed amount of fluid (10, 20, 30 µL) by means of a hydrophilic polymer tip, thus allowing an accurate blood collection directly from a fingerprick, regardless of the blood density. VAMS thus avoids HCT-depending issues and makes point-of-care sampling and self-sampling even more feasible [23,24,25]. After drying/storage at room temperature, the VAMS device tip can undergo extraction with different pure solvents or mixtures and different extraction means [26]. Despite its relatively recent introduction, VAMS technology has been successfully applied to several analytical challenges (pharmacokinetics [27], therapeutic drug monitoring [24,28], analysis of drug of abuse [29]) and to different biological fluids [30]. VAMS has also been applied to the discovery of protein biomarkers [31] and to targeted metabolomics [32,33]. Nevertheless, to the best of our knowledge, this innovative and promising approach has never been studied before for untargeted lipidomic analysis. In this proof-of-concept study, untargeted lipidomic profiling in whole blood microsamples has been carefully investigated by using VAMS microsampling and subsequent extraction of analytes with different solvents and mixtures. VAMS has also been compared to DBS and classic fluid blood under the same conditions. The recovered lipids were analysed by high-resolution UHPLC-MS/MS, and the corresponding datasets were analysed and compared by using multivariate data analysis. The aim of this work was to evaluate the potential of VAMS as a viable and promising alternative strategy for the sampling of microvolumes of whole blood for untargeted lipidomic analysis.

## 2. Results and Discussion

**Figure 1:**
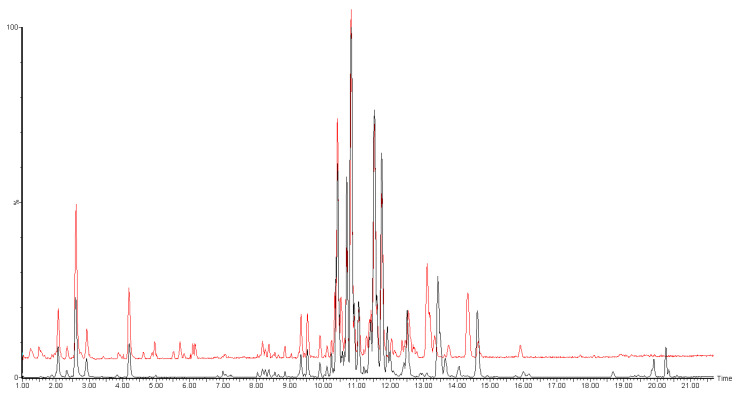
Representative overlapped chromatograms of untargeted LC-MS/MS analysis of blood lipidome, reporting both ESI+ (black) and ESI− (red) traces. ESI− trace was offset by 5% of the axis for better clarity.

In this work, we aimed to evaluate the best combination of solid support (DBS or VAMS) and extraction solvent (IPA, MeOH, MeOH/CHCl_3_ and MeOH/MTBE) for the sampling and untargeted analysis of the whole blood lipidome. For this purpose, we tested all the possible combinations, in triplicates. As a reference, we also extracted lipids from liquid, intact blood, without any adsorption process. For all the tests, the LC-MS/MS conditions were kept fixed, using the well-established isopropanol gradient on a reversed phase column, coupled with detection by high-resolution MS. Each sample was acquired in both positive and negative ion modes. Figure 1 shows representative chromatograms from an untargeted analysis of the blood lipidome. 

**Figure 2:**
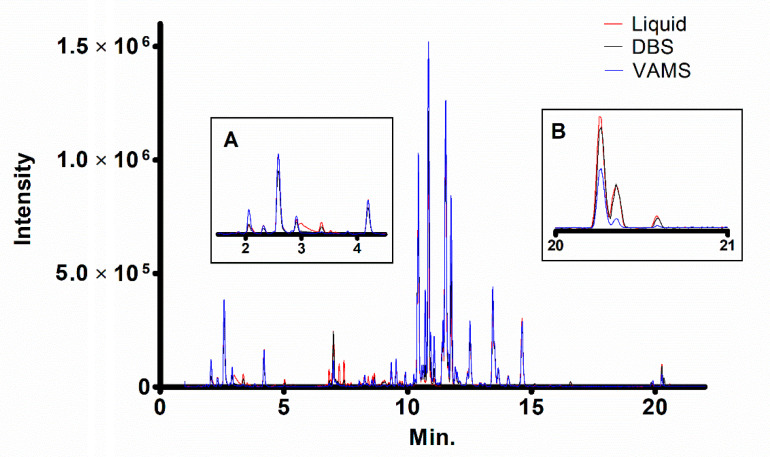
Overlapped ESI+ chromatograms of lipids extracted from 20 μL of human blood with 9:1 MeOH/CHCl_3_ in liquid form, or obtained through dried blood spot (DBS) or volumetric absorptive microsampling (VAMS) methods. Insets report the magnification of the initial (**A**) and final (**B**) part of the chromatogram.

In each comparison, we first fixed the extraction solvent, and then compared the two different microsampling techniques (VAMS and DBS) with the corresponding extraction of liquid blood performed with that solvent. As an example, Figure 2 shows the overlapped ESI+ chromatograms of VAMS, DBS, or fluid blood (20 µL) extracted with 9:1 MeOH/CHCl_3_.

**Figure 3:**
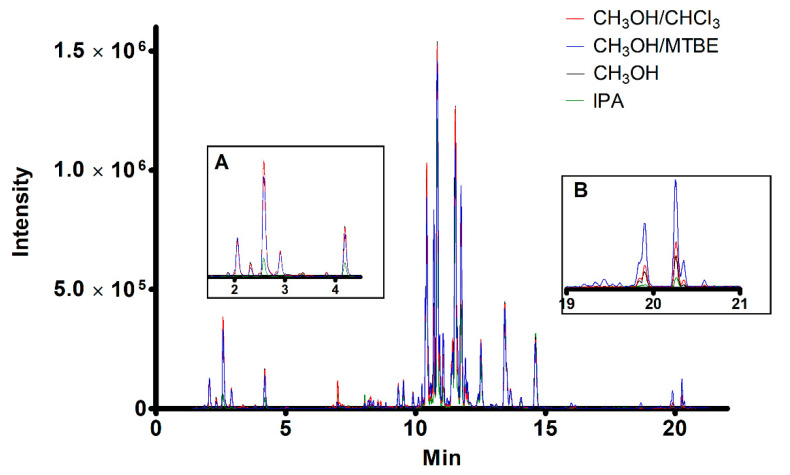
Overlapped ESI+ chromatograms of lipids recovered from VAMS (20 μL of human blood) using the four solvents. Insets report the magnification of the initial (**A**) and final (**B**) part of the chromatogram.

From a general inspection of the chromatograms, the three overall profiles obtained using the same solvent appear quite comparable, thus implying that DBS and VAMS yield similar lipid recoveries compared to liquid blood. We then fixed the solid support and compared the solvents used to recover the lipids. As an example, Figure 3 shows the overlapped chromatogram of the lipidome recovered from VAMS (20 μL of human whole blood) by using the four solvents.

With the purpose of finding a metric to evaluate the performances of each of the conditions, we then selectively extracted the data on a set of 15 representative lipids belonging to five different lipid categories and spanning over a broad range of logP and retention time values. Table 1 reports the details of the chosen individual lipids. The Supplementary Materials report all the experimental evidence related to the detection of these molecules.

These lipids are representatives of the lipidomic chemical space, and thus serve as indicators of the ability of each support/solvent combination to cover the maximum extension of the blood lipidome for untargeted studies. Furthermore, since these lipids are representative of the most important and abundant lipid categories of the mammalian lipidome, their abundance in each experiment serves as a benchmark to evaluate the performance of each system in the profiling of the whole blood lipidome for biomedical applications in general. The corresponding data matrix, reporting the peak area of each individual lipid in all the samples, is available as Supplementary File 1. We then used principal component analysis (PCA) to compare how the four solvents performed in extracting lipids from the three conditions (fluid blood, DBS and VAMS). The resulting scores and loadings plots are reported in Supplementary Figures S1, S2 and S3 (in Supplementary Powerpoint File), respectively.

**Figure 4:**
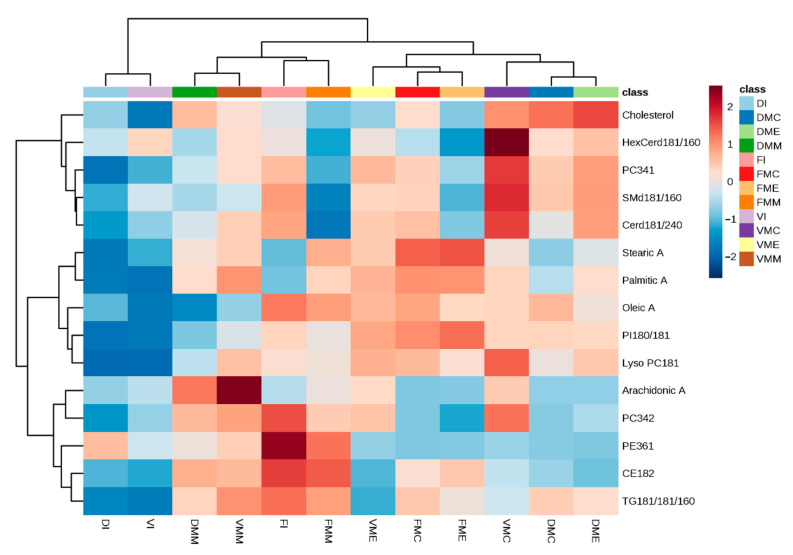
Heatmap illustrating the recoveries of the 15 benchmark lipids from the three conditions (VAMS, DBS and liquid form) using the four solvents (IPA, MeOH, MeOH/CHCl_3_ or MeOH/MTBE). Legend refers to the condition tested (D, DBS; F, fluid blood; and V, VAMS) and the corresponding solvent used (I, isopropanol; MC, methanol/chloroform; ME, methanol; MM, methanol/MTBE).

When extracting lipids from intact fluid blood, MeOH/MTBE and IPA behave in similar ways, but distinct from MeOH and MeOH/CHCl_3_ (Figure S1). When extracting lipids from DBS, MeOH/CHCl_3_, IPA and MeOH show overall similar recoveries, while MeOH/MTBE shows a significantly different lipid profile (Figure S2). As illustrated in Figure S3, for VAMS, each solvent except MeOH and MeOH/CHCl_3_, shows a distinct composition of the 15 benchmark lipids. Furthermore, the overall variability in lipid recovery appears to be dramatically lower from VAMS than from the other two conditions, as indicated by the 95% confidence area indicated in the graph. In order to obtain a general picture of lipid recovery from all the tested conditions, we prepared a heatmap (Figure 4) summarising the recovery of all the 15 benchmark lipids from each of the three conditions or supports (VAMS, DBS or liquid blood), using each of the four solvents.

**Figure 5:**
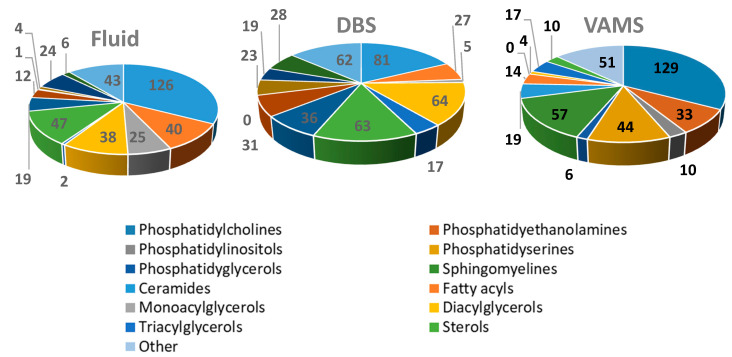
Pie charts reporting the global composition of the individually identified lipids for the three conditions using their best extraction solvents: fluid blood with IPA, DBS with MeOH/CHCl_3_ and VAMS with MeOH/MTBE.

As illustrated by the heatmap, IPA does not efficiently recover lipids from DBS and VAMS, although it is a good choice for intact fluid blood. When the sample is DBS, MeOH efficiently extracts free cholesterol and sphingolipids, slightly better than MeOH/CHCl_3_ does. For less polar lipids, such as TAG and CE, MeOH/MTBE works better on DBS. VAMS works very well for a broad range of lipids (sphingolipids, free cholesterol, free fatty acids, phospholipids) when extracted with MeOH/CHCl_3_ and MeOH/MTBE. When pure MeOH is used on this support, the recovery of apolar lipids is reduced. From a general perspective, VAMS appears to be more flexible and efficient than DBS for a general profiling of many lipid classes. As above indicated, the list of 15 benchmark lipids were arbitrarily selected to provide a general overview of the performance of different solid supports over a broad selection of lipid families. Different benchmarks can be chosen by simply interrogating the full RAW dataset related to this study, publicly shared and available through Metabolights [34]. As a final check, we compared the three best performing conditions (fluid blood with IPA, VAMS with MeOH/MTBE, and DBS with MeOH/CHCl_3_) in terms of total number of individual lipid species positively identified, by using the automated lipid identification feature of Lipostar software. A total of 387, 456 and 394 individual lipids were identified in the three conditions, respectively, with 110 lipids observed in all three groups. The details of lipid annotation are reported in Supplementary File 2. While accounting for annotation uncertainties, these numbers are quite consistent with previous initiatives [35], reporting the consistent (multi-laboratory) observation of 226 lipid species. Figure 5 reports the corresponding pie charts of the composition per class of the identified lipids.

## 3. Materials and Methods

### 3.1. Solvents and Instrumentation

Acetonitrile (ACN), chloroform (CHCl_3_), methanol (MeOH), ammonium formate, and tert-butyl methyl ether (MTBE) were of HPLC or LC-MS grade, and Whatman 903 protein saver cards were purchased from Sigma Aldrich (Saint Louis, MO, USA). Isopropanol (IPA) was purchased from VWR (Radnor, PA, USA). Ultrapure water (18.2 MΩ cm) was obtained by means of MilliQ apparatus from Millipore (Milford, MS, USA). VAMS devices (20 µL) were purchased from Neoteryx (Torrance, CA, USA) under the brand name of Mitra^®^. All LC-MS instruments, the column, and software were from Waters Inc. (Milford, MA, USA).

### 3.2. Biological Sample Collection

The whole blood sample was provided by a healthy donor not enrolled in any study, collected by venipuncture and stored in a test tube containing anticoagulant (heparin) at 4 °C for 2 h prior to sampling. A volume of 20 µL was accurately measured by micropipette and transferred to microcentrifuge tubes to prepare fluid blood samples. For DBS, an accurate volume of 20 µL was deposited on a 903 protein saver card (Whatman 903). The cards were dried at RT for 1 h and stored in the dark in sealed plastic bags containing silica gel packets, for 1 month at most until analysis. For VAMS, the whole blood sample was adsorbed onto a fixed 20 µL polymeric tip. The devices were then dried at RT in their dedicated clamshell package for 1 h and stored at RT in the dark for 1 month at most until analysis. Safety Declaration: no unexpected or new significant hazards or risks are associated with the reported work.

### 3.3. Extraction of Lipids from Blood Samples

All samples were processed following the same pre-treatment procedure. The fluid blood, DBS and VAMS tip were extracted in microcentrifuge tubes with 500 µL of different solvents (100% IPA, 100% MeOH) and solvent mixtures (50:50 MeOH/MTBE *v/v*, 90:10 MeOH/CHCl_3_ *v/v*) by means of ultrasound-assisted extraction (UAE) for 10 min and subsequent vortex-assisted extraction (VAE) for 2 min. For DBS, the whole spotted area was excised and extracted. For VAMS, the whole tip was detached from the plastic handler and extracted. After centrifugation at 4000 rpm for 5 min at 4 °C, the supernatant was dried under a gentle nitrogen gas stream and stored in microcentrifuge tubes at RT until analysis. Each extraction mode and condition was tested in triplicate. On the day of analysis, the dried samples were dissolved in 80 µL of a mixture composed of 90:10 MeOH/CHCl_3_ *v/v*. After VAE for 5 min and centrifugation for 5 min at 4 °C, the samples were transferred into glass vials for UHPLC-ESI (+/−)-QTOF-MS analyses. Seven quality control (QC) samples were prepared by pooling together 5 µL aliquots from each of the samples. Blank samples were prepared by mimicking the same extraction procedures with empty tubes.

### 3.4. LC-MS/MS Analysis

Lipid samples were loaded onto an Acquity UPLC system coupled to a Synapt G2 QTOF Mass Spectrometer (all LC-MS instruments and columns were purchased from Waters Inc. Milford, MA, USA). The chromatographic separation was performed on a reversed-phase CSH column (2.1 mm × 100 mm, 1.8 µm) maintained at 55 °C at a flow rate of 0.4 mL/min. The mobile phases consisted of A: 10 mM ammonium formate in 60:40 ACN/H_2_O *v/v*; and B: 10 mM ammonium formate in 90:10 IPA/ACN *v/v*. The gradient composition was 15% B for 0–1 min, 60% B for 1–10 min, 75% B for 10–18 min. Then, solvent B was brought to 100% for 18–21.5 min, followed by 100% B isocratic step for 21.5–23 min and reconditioning to 15% B. The total run time was 25 min. The injection volume was 5 µL and the tray temperature was set at 6 °C. The untargeted lipidomic analysis was performed in both positive and negative ion modes. The source temperature was set to 90 °C, and desolvation temperature was set to 400 °C. Desolvation and cone gas flows (N_2_) were set to 800 and 50 L/h, respectively. Data were acquired in MS^e^. The scan rate was set to 0.3 s per spectrum; the scan range was set to 50 to 1200 *m/z*. Leucine enkephalin (2 ng/mL) was infused as lock mass for real-time spectra recalibration. The UHPLC-MS/MS system and the column were purchased from Waters Inc. (Milford, MA USA). A system suitability test, aiming to assess peak shape, retention time and instrument sensitivity, was preliminarily performed by running a reference sample, consisting of a mixture of 1 μM authentic standards dissolved in 9:1 MeOH/CHCl_3_ *v/v*. Each sample was acquired twice (ESI+ and ESI−). The samples were acquired in a random order, intermixed with QCs and solvent samples (90:10 MeOH/CHCl_3_ *v/v*).

### 3.5. Data Analysis

QC samples were used to evaluate calibration stability over time (mass accuracy never exceeded 3 ppm) and retention time reproducibility (which never exceeded 0.1 min). High resolution MS and MS/MS data were used to confidently assign the structure of the 15 benchmark lipids. First, the *m/z* value of the precursor ion was searched against LipidMaps database [36] to obtain a putative ID. The corresponding MS/MS spectrum, in combination with the LipidMaps dedicated bioinformatics tools, was used to confirm the lipid ID. The peak areas for each of the 15 benchmark lipids was then integrated using Targetlynx software (version 4.1) (Waters (Milford, MA, USA)), by extracting the ion current of the corresponding precursor ion at the corresponding retention time (0.01 *m/z* and 0.1 min were selected as maximum tolerance values for the mass and retention time values, respectively). The software was set to automatically deconvolve the features by detecting their most common adducts [M+H]^+^, [M+Na]^+^, [M+NH_4_]^+^, [M+K]^+^, [M–H_2_O+H]^+^ for ESI+, and [M–H]^−^, [M+HCCO]^−^ for ESI−. The integrated peak area for each lipid was then verified by visual inspection in each sample. The obtained data matrix (36 samples × 15 lipids) was then analysed using the Metaboanalyst [37] software suite to perform principal component analysis and produce heatmaps. The peak areas were first normalized by the total peak area of each sample, then log-transformed and Pareto-scaled. For heatmap generation, the Euclidean distance was used with Ward algorithm for cluster analysis [38]. Lipostar software [39] (version 1.0.5, from Molecular Horizons) was used for automated lipid identification: the LipidMaps lipid library and Lipostar MS/MS fragmentation rules [40] were applied for feature identification. A maximum tolerance of 0.01 amu mass accuracy was used for both MS and MS/MS spectra.

### 3.6. Data Availability

All the RAW datafiles related to this study were openly shared through the Metabolights data repository [34], and are thus publicly available (dataset unique identifier: MTBLS1200).

## 4. Conclusions

Based on our experimental evidence, we can confidently state that both DBS and VAMS represent very interesting options as solid supports for the sampling, storage and analysis of the blood lipidome for subsequent untargeted lipidomics analysis. Although this was already demonstrated by Aristizabal Henao et al. [19] for DBS, to the best of our knowledge, this is the first time that the potential of VAMS for untargeted lipidomic applications has been explored. We are aware that the present work is limited to a series of repeated tests on the blood of a single donor. Furthermore, additional studies involving the use of reference standards will be needed to more precisely quantify the recovery of lipids for targeted studies. In particular, the use of deuterated internal standards will also help to assess the matrix effects that each solid support has on individual lipids classes. Indeed, because we believe that the current research represents a first, proof-of-concept study on a potentially totally novel approach to untargeted lipidomics, to be soon supported by further studies, we purportedly removed the interindividual variability from our work, to focus on the comparisons between the different studies. Quite surprisingly, when the lipid content was extracted with an appropriate solvent, VAMS enabled the recovery and analysis of more than 450 individual lipids, with a particular enrichment in sphingolipids (Figure 5). In our experiments, the performances of VAMS, in terms of number of individual lipid species recovered and profiled, significantly surpassed those of DBS. Now that VAMS has been demonstrated to be a reliable, although at the moment more expensive, alternative to DBS, new, dedicated studies will perhaps explore other possible solvents for VAMS, with the aim to enrich the lipid content recovery even further. In this perspective, our results will be extremely useful for the targeted analysis of lipids supported by VAMS. Furthermore, our paper also represents a systematic investigation of the lipid chemical space currently accessible by both DBS and VAMS.

## Figures and Tables

**Table 1:** Lipid species selected for recovery evaluation tests.

N	Category	Lipid	Molecular Formula	LogP	*m/z*	Adduct	Min.
1	Fatty Acid	Arachidonic Acid (20:4)	C_20_H_31_O_2_	6.22	303.23	[M–H]^−^	4.6
2	Fatty Acid	Oleic Acid (18:1)	C_18_H_33_O_2_	6.11	281.24	[M–H]^−^	6.1
3	Fatty Acid	Stearic Acid (18:0)	C_18_H_35_O_2_	6.33	283.20	[M–H]^−^	7.6
4	Fatty Acid	Palmitic Acid (16:0)	C_16_H_31_O_2_	5.55	255.23	[M–H]^−^	5.8
5	Phospholipid	PI (18:0/18:1)	C_45_H_85_O_13_P	11.81	863.57	[M–H]^−^	11.2
6	Phospholipid	Lyso PC (18:1)	C_26_H_51_NO_7_P	6.56	520.34	[M+H]^+^	2.0
7	Phospholipid	PC (34:2)	C_42_H_79_NO_8_P	12.37	756.55	[M+H]^+^	10.3
8	Phospholipid	PC (34:1)	C_42_H_81_NO_8_P	12.59	758.57	[M+H]^+^	10.8
9	Phospholipid	PE (36:1)	C_41_H_81_NO_8_P	13.43	746.57	[M+H]^+^	11.0
10	Sphingolipid	SM (d18:1/16:0)	C_39_H_80_N_2_O_6_P	11.21	703.57	[M+H]^+^	10.4
11	Sphingolipid	HexCer (d18:1/16:0)	C_40_H_78_NO_8_	9.96	700.57	[M+H]^+^	10.8
12	Sphingolipid	Cer (d18:1/24:0)	C_42_H_84_NO_3_	13.54	650.64	[M+H]^+^	16.1
13	Sterol	Cholesterol	C_27_H_44_	7.68	369.35	[M–H_2_O+H]^+^	10.3
14	Sterol	CE (18:2)	C_45_H_77_O_2_	14.04	666.62	[M+NH_4_]^+^	19.8
15	Triacylglycerol	TG (16:0/18:1/18:1)	C_55_H_106_NO_6_	18.4	876.80	[M+NH_4_]^+^	20.2

## Data Availability

All the data related to this paper are publicly available at: www.ebi.ac.uk/metabolights/MTBLS1200.

## Supplementary Materials

The following are available online, Supplementary Excel File 1, Supplementary Excel File 2 and Supplementary Powerpoint File.

## Sample Availability

Samples of the compounds are not available from the authors.

## References

[1] Wenk M.R. (2005). The emerging field of lipidomics. Nat. Rev. Drug Discov..

[2] Afshinnia F., Rajendiran T.M., Wernisch S., Soni T., Jadoon A., Karnovsky A., Michailidis G., Pennathur S. (2018). Lipidomics and Biomarker Discovery in Kidney Disease. Semin. Nephrol..

[3] Herzog K., Pras-Raves M.L., Ferdinandusse S., Vervaart M.A.T., Luyf A.C.M., Van Kampen A.H.C., Wanders R.J.A., Waterham H.R., Vaz F.M. (2017). Plasma lipidomics as a diagnostic tool for peroxisomal disorders. J. Inherit. Metab. Dis..

[4] Wood P.L., Cebak J.E. (2018). Lipidomics biomarker studies: Errors, limitations, and the future. Biochem. Biophys. Res. Commun..

[5] Yan F., Zhao H., Zeng Y. (2018). Lipidomics: A promising cancer biomarker. Clin. Transl. Med..

[6] Burla B., Arita M., Arita M., Bendt A.K., Gassiot A.C., Dennis E.A., Ekroos K., Han X., Ikeda K., Liebisch G. (2018). MS-based lipidomics of human blood plasma: A community-initiated position paper to develop accepted guidelines. J. Lipid Res..

[7] Hu T., Zhang J. (2018). Mass-spectrometry-based lipidomics. J. Sep. Sci..

[8] Zalloua P.A., Kadar H., Hariri E., Farraj L.A., Brial F., Hedjazi L., Le Lay A., Colleu A., Dubus J., Touboul D. (2019). Untargeted Mass Spectrometry Lipidomics identifies correlation between serum sphingomyelins and plasma cholesterol. Lipids Health Dis..

[9] Cajka T., Fiehn O. (2014). Comprehensive analysis of lipids in biological systems by liquid chromatography-mass spectrometry. TrAC Trends Anal. Chem..

[10] Baglai A., Gargano A.F., Jordens J., Mengerink Y., Honing M., van der Wal S., Schoenmakers P.J. (2017). Comprehensive lipidomic analysis of human plasma using multidimensional liquid- and gas-phase separations: Two-dimensional liquid chromatography-mass spectrometry vs. liquid chromatography-trapped-ion-mobility-mass spectrometry. J. Chromatogr. A.

[11] Rampler E., Criscuolo A., Zeller M., El Abiead Y., Schoeny H., Hermann G., Sokol E., Cook K., Peake D.A., Delanghe B. (2018). A Novel Lipidomics Workflow for Improved Human Plasma Identification and Quantification Using RPLC-MSn Methods and Isotope Dilution Strategies. Anal. Chem..

[12] Song J., Liu X., Wu J., Meehan M.J., Blevitt J.M., Dorrestein P.C., Milla M.E. (2013). A highly efficient, high-throughput lipidomics platform for the quantitative detection of eicosanoids in human whole blood. Anal. Biochem..

[13] Yin P., Lehmann R., Xu G. (2015). Effects of pre-analytical processes on blood samples used in metabolomics studies. Anal. Bioanal. Chem..

[14] Becker S., Röhnike S., Empting S., Haas D., Mohnike K., Beblo S., Mütze U., Husain R.A., Thiery J., Ceglarek U. (2015). LC-MS/MS-based quantification of cholesterol and related metabolites in dried blood for the screening of inborn errors of sterol metabolism. Anal. Bioanal. Chem..

[15] Jacob M., Malkawi A., Albast N., Al Bougha S., Lopata A., Dasouki M., Rahman A.M.A. (2018). A targeted metabolomics approach for clinical diagnosis of inborn errors of metabolism. Anal. Chim. Acta.

[16] Koulman A., Prentice P., Wong M.C.Y., Matthews L., Bond N.J., Eiden M., Griffin J.L., Dunger D.B. (2014). The development and validation of a fast and robust dried blood spot based lipid profiling method to study infant metabolism. Metabolomics.

[17] Dittakavi S., Jat R.K., Mullangi R. (2019). Quantitative analysis of enasidenib in dried blood spots of mouse blood using an increased-sensitivity LC-MS/MS method: Application to a pharmacokinetic study. Biomed. Chromatogr..

[18] Björkesten J., Enroth S., Shen Q., Wik L., Hougaard D.M., Cohen A.S., Sörensen L., Giedraitis V., Ingelsson M., Larsson A. (2017). Stability of Proteins in Dried Blood Spot Biobanks. Mol. Cell. Proteom..

[19] Henao J.J.A., Metherel A.H., Smith R.W., Stark K.D. (2016). Tailored Extraction Procedure Is Required to Ensure Recovery of the Main Lipid Classes in Whole Blood When Profiling the Lipidome of Dried Blood Spots. Anal. Chem..

[20] Cozma C., Iurașcu M.-I., Eichler S., Hovakimyan M., Brandau O., Zielke S., Böttcher T., Giese A.-K., Lukas J., Rolfs A. (2017). C26-Ceramide as highly sensitive biomarker for the diagnosis of Farber Disease. Sci. Rep..

[21] Kyle J.E., Casey C.P., Stratton K.G., Zink E.M., Kim Y.-M., Zheng X., Monroe M.E., Weitz K.K., Bloodsworth K.J., Orton D.J. (2017). Comparing identified and statistically significant lipids and polar metabolites in 15-year old serum and dried blood spot samples for longitudinal studies. Rapid Commun. Mass Spectrom..

[22] Gao F., McDaniel J., Chen E.Y., Rockwell H.E., Drolet J., Vishnudas V.K., Tolstikov V., Sarangarajan R., Narain N.R., Kiebish M.A. (2017). Dynamic and temporal assessment of human dried blood spot MS/MS(ALL) shotgun lipidomics analysis. Nutr. Metab..

[23] Denniff P., Spooner N. (2014). Volumetric Absorptive Microsampling: A Dried Sample Collection Technique for Quantitative Bioanalysis. Anal. Chem..

[24] Velghe S., Stove C.P. (2018). Volumetric absorptive microsampling as an alternative tool for therapeutic drug monitoring of first-generation anti-epileptic drugs. Anal. Bioanal. Chem..

[25] Verougstraete N., Lapauw B., Van Aken S., Delanghe J., Stove C., Stove V. (2017). Volumetric absorptive microsampling at home as an alternative tool for the monitoring of HbA(1c) in diabetes patients. Clin. Chem. Lab. Med..

[26] Protti M., Mandrioli R., Mercolini L. (2019). Tutorial: Volumetric absorptive microsampling (VAMS). Anal. Chim. Acta.

[27] Kita K., Noritake K., Mano Y. (2018). Application of a Volumetric Absorptive Microsampling Device to a Pharmacokinetic Study of Tacrolimus in Rats: Comparison with Wet Blood and Plasma. Eur. J. Drug Metab. Pharmacokinet..

[28] Protti M., Catapano M.C., Dekel B.G.S., Rudge J., Gerra G., Somaini L., Mandrioli R., Mercolini L. (2018). Determination of oxycodone and its major metabolites in haematic and urinary matrices: Comparison of traditional and miniaturised sampling approaches. J. Pharm. Biomed. Anal..

[29] Protti M., Rudge J., Sberna A.E., Gerra G., Mercolini L. (2017). Dried haematic microsamples and LC-MS/MS for the analysis of natural and synthetic cannabinoids. J. Chromatogr. B Anal. Technol. Biomed. Life Sci..

[30] Mercolini L., Protti M., Catapano M.C., Rudge J., Sberna A.E. (2016). LC-MS/MS and volumetric absorptive microsampling for quantitative bioanalysis of cathinone analogues in dried urine, plasma and oral fluid samples. J. Pharm. Biomed. Anal..

[31] Van den Broek I., Fu Q., Kushon S., Kowalski M.P., Millis K., Percy A., Holewinski R.J., Venkatraman V., Van Eyk J.E. (2017). Application of volumetric absorptive microsampling for robust, high-throughput mass spectrometric quantification of circulating protein biomarkers. Clin. Mass Spectrom..

[32] Kok M.G., Nix C., Nys G., Fillet M. (2019). Targeted metabolomics of whole blood using volumetric absorptive microsampling. Talanta.

[33] Volani C., Caprioli G., Calderisi G., Sigurdsson B.B., Rainer J., Gentilini I., Hicks A.A., Pramstaller P.P., Weiss G., Smarason S.V. (2017). Pre-analytic evaluation of volumetric absorptive microsampling and integration in a mass spectrometry-based metabolomics workflow. Anal. Bioanal. Chem..

[34] Kale N.S., Haug K., Conesa P., Jayseelan K., Moreno P., Rocca-Serra P., Nainala V.C., Spicer R.A., Williams M., Philippe R.-S. (2016). MetaboLights: An Open-Access Database Repository for Metabolomics Data. Curr. Protoc. Bioinform..

[35] Bowden J.A., Heckert A., Ulmer C.Z., Jones C.M., Koelmel J.P., Abdullah L., Ahonen L., Alnouti Y., Armando A.M., Asara J.M. (2017). Harmonizing lipidomics: NIST interlaboratory comparison exercise for lipidomics using SRM 1950-Metabolites in Frozen Human Plasma. J. Lipid Res..

[36] Fahy E., Sud M., Cotter D., Subramaniam S. (2007). LIPID MAPS online tools for lipid research. Nucleic Acids Res..

[37] Xia J., Wishart D.S. (2016). Using MetaboAnalyst 3.0 for Comprehensive Metabolomics Data Analysis. Curr. Protoc. Bioinform..

[38] Chong J., Xia J. (2018). MetaboAnalystR: An R package for flexible and reproducible analysis of metabolomics data. Bioinformatics.

[39] Goracci L., Tortorella S., Tiberi P., Pellegrino R.M., Di Veroli A., Valeri A., Cruciani G. (2017). Lipostar, a Comprehensive Platform-Neutral Cheminformatics Tool for Lipidomics. Anal. Chem..

[40] La Barbera G., Antonelli M., Cavaliere C., Cruciani G., Goracci L., Montone C.M., Piovesana S., Laganà A., Capriotti A.L. (2018). Delving into the Polar Lipidome by Optimized Chromatographic Separation, High-Resolution Mass Spectrometry, and Comprehensive Identification with Lipostar: Microalgae as Case Study. Anal. Chem..

