# Supplementary material for: Volumetric Absorptive Microsampling of Blood for Untargeted Lipidomics
Source: Molecules. 2021 Jan 7;26(2):262. doi: 10.3390/molecules26020262 (PMC7825730; doi:10.3390/molecules26020262)
Supplement: Supplementary file 1 [file molecules-26-00262-s001.zip › molecules-1054610-revised-1-SM/Supplementary File.pptx]

## Slide 1
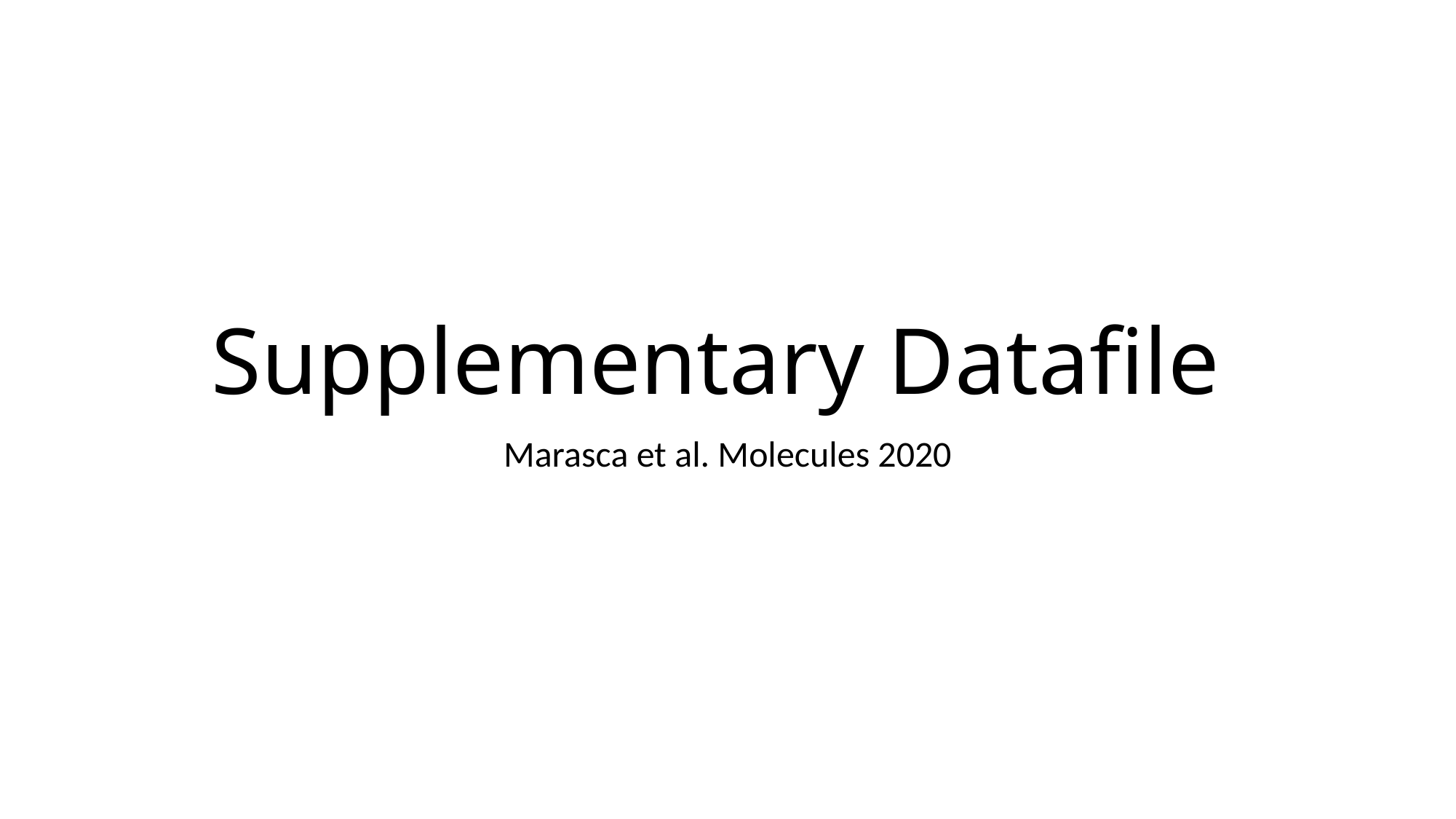

# Supplementary Datafile
Marasca et al. Molecules 2020

## Slide 2
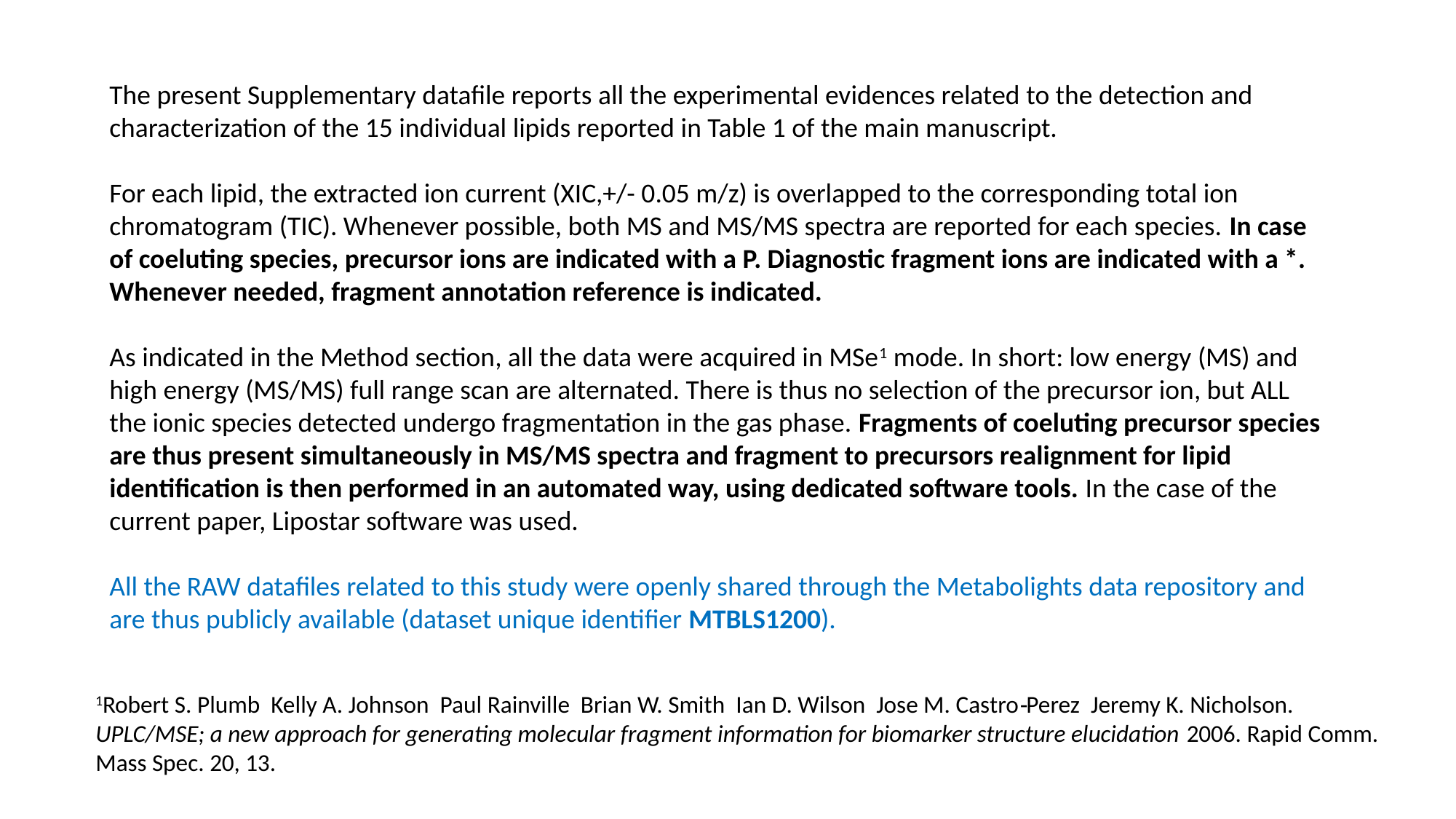

The present Supplementary datafile reports all the experimental evidences related to the detection and characterization of the 15 individual lipids reported in Table 1 of the main manuscript.
For each lipid, the extracted ion current (XIC,+/- 0.05 m/z) is overlapped to the corresponding total ion chromatogram (TIC). Whenever possible, both MS and MS/MS spectra are reported for each species. In case of coeluting species, precursor ions are indicated with a P. Diagnostic fragment ions are indicated with a *.
Whenever needed, fragment annotation reference is indicated.
As indicated in the Method section, all the data were acquired in MSe1 mode. In short: low energy (MS) and high energy (MS/MS) full range scan are alternated. There is thus no selection of the precursor ion, but ALL the ionic species detected undergo fragmentation in the gas phase. Fragments of coeluting precursor species are thus present simultaneously in MS/MS spectra and fragment to precursors realignment for lipid identification is then performed in an automated way, using dedicated software tools. In the case of the current paper, Lipostar software was used.
All the RAW datafiles related to this study were openly shared through the Metabolights data repository and are thus publicly available (dataset unique identifier MTBLS1200).
1Robert S. Plumb Kelly A. Johnson Paul Rainville Brian W. Smith Ian D. Wilson Jose M. Castro‐Perez Jeremy K. Nicholson. UPLC/MSE; a new approach for generating molecular fragment information for biomarker structure elucidation 2006. Rapid Comm. Mass Spec. 20, 13.

## Slide 3
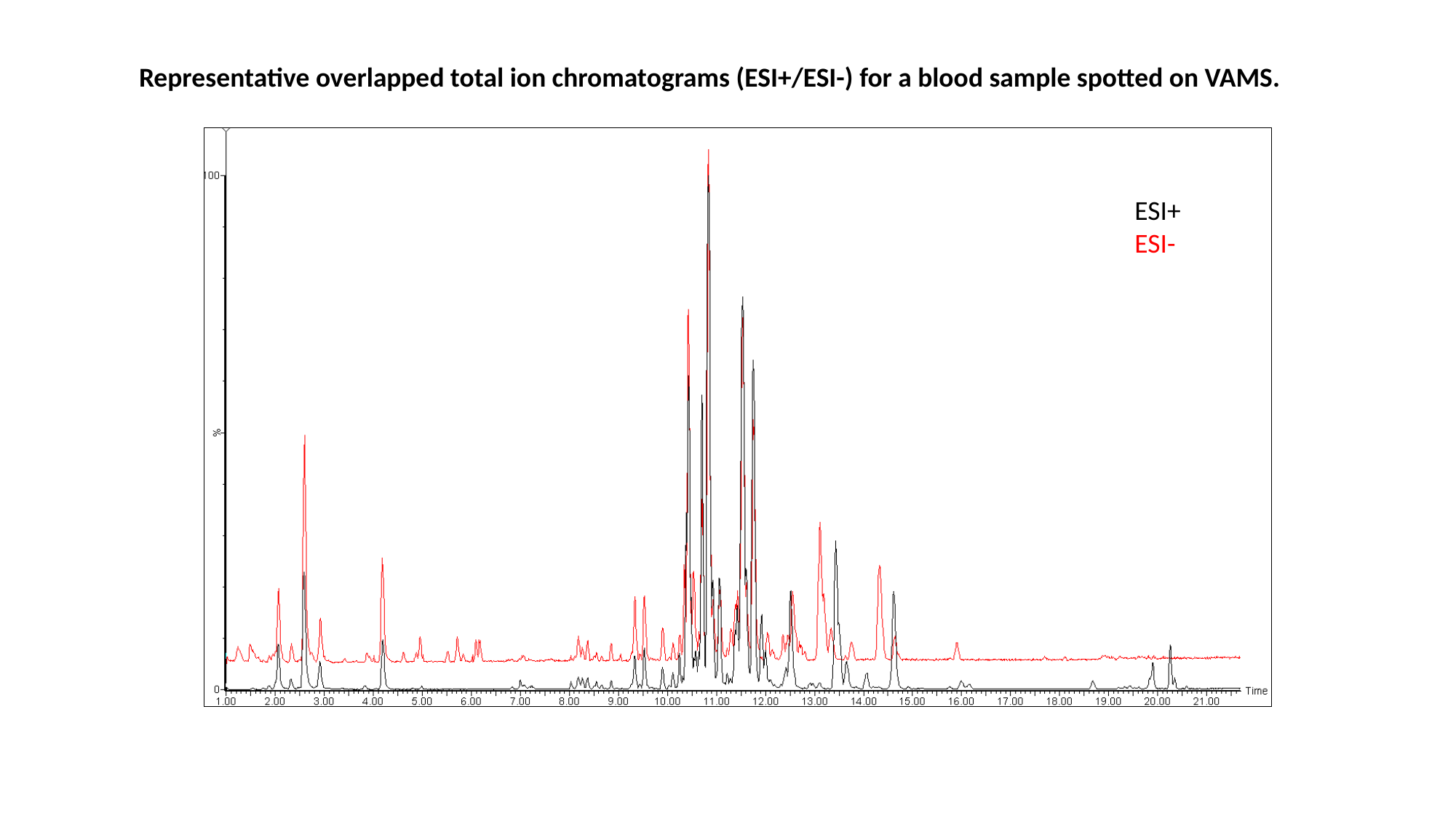

Representative overlapped total ion chromatograms (ESI+/ESI-) for a blood sample spotted on VAMS.
ESI+
ESI-

## Slide 4
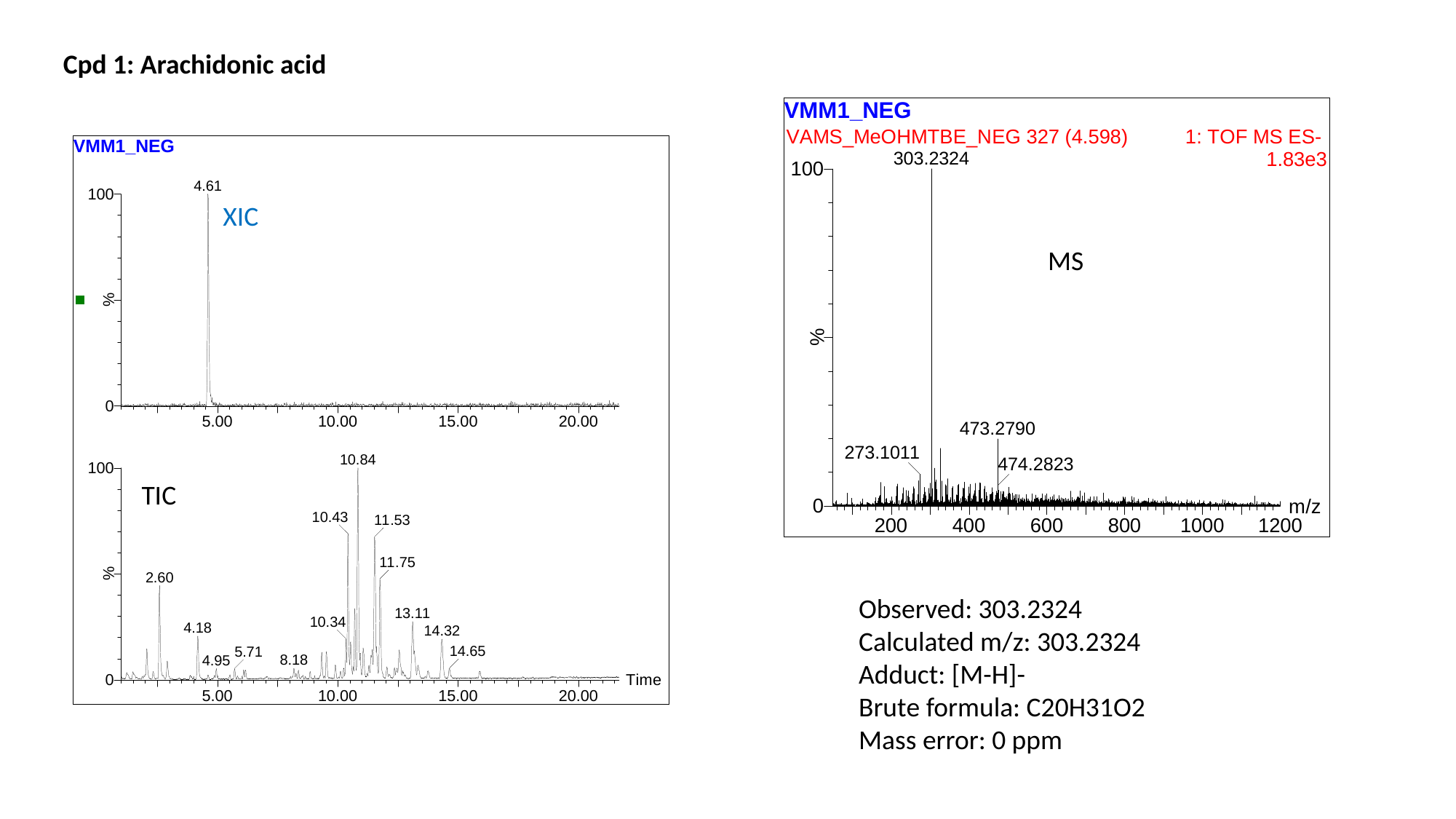

Cpd 1: Arachidonic acid
XIC
MS
TIC
Observed: 303.2324
Calculated m/z: 303.2324
Adduct: [M-H]-
Brute formula: C20H31O2
Mass error: 0 ppm

## Slide 5
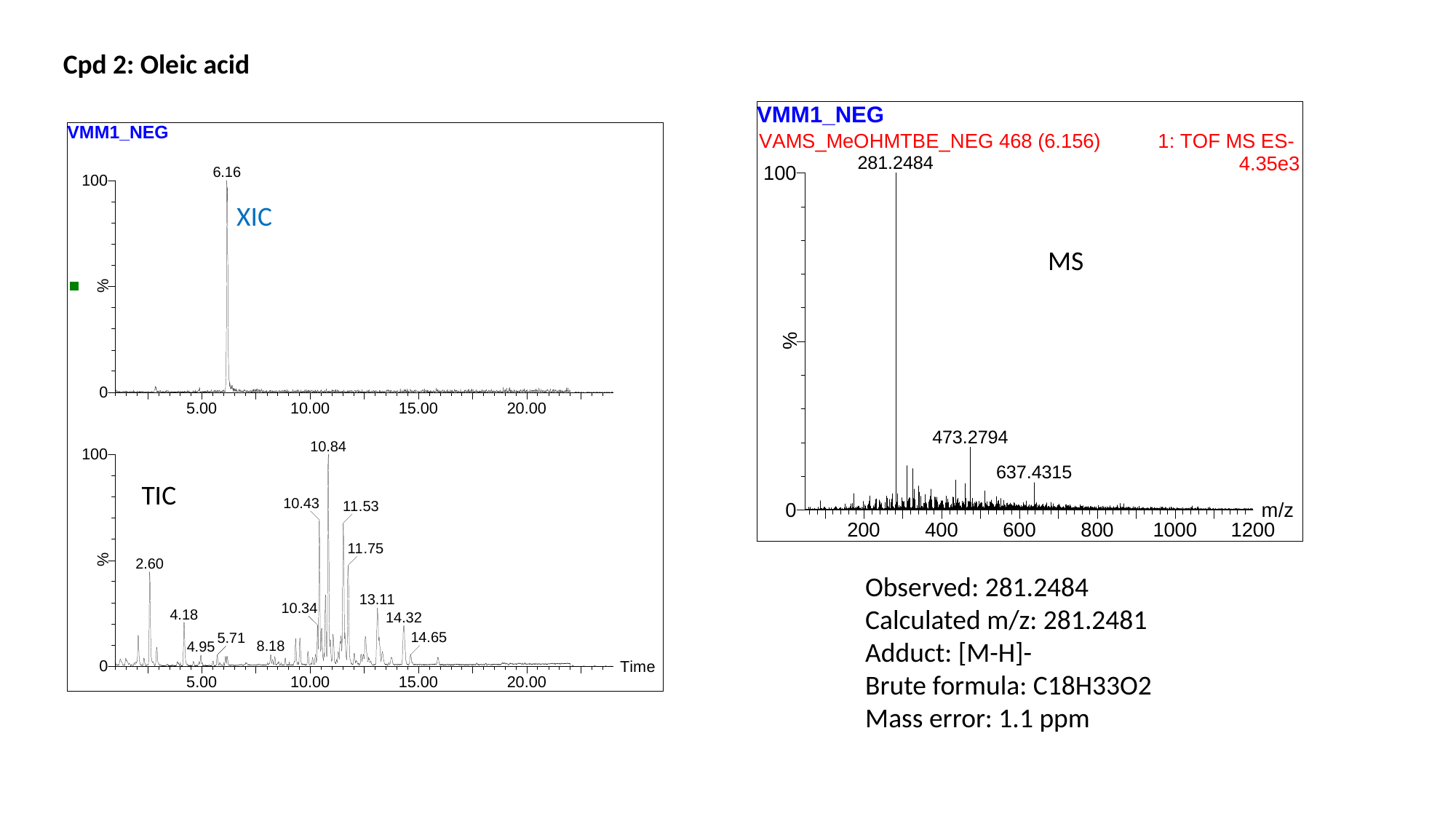

Cpd 2: Oleic acid
XIC
MS
TIC
Observed: 281.2484
Calculated m/z: 281.2481
Adduct: [M-H]-
Brute formula: C18H33O2
Mass error: 1.1 ppm

## Slide 6
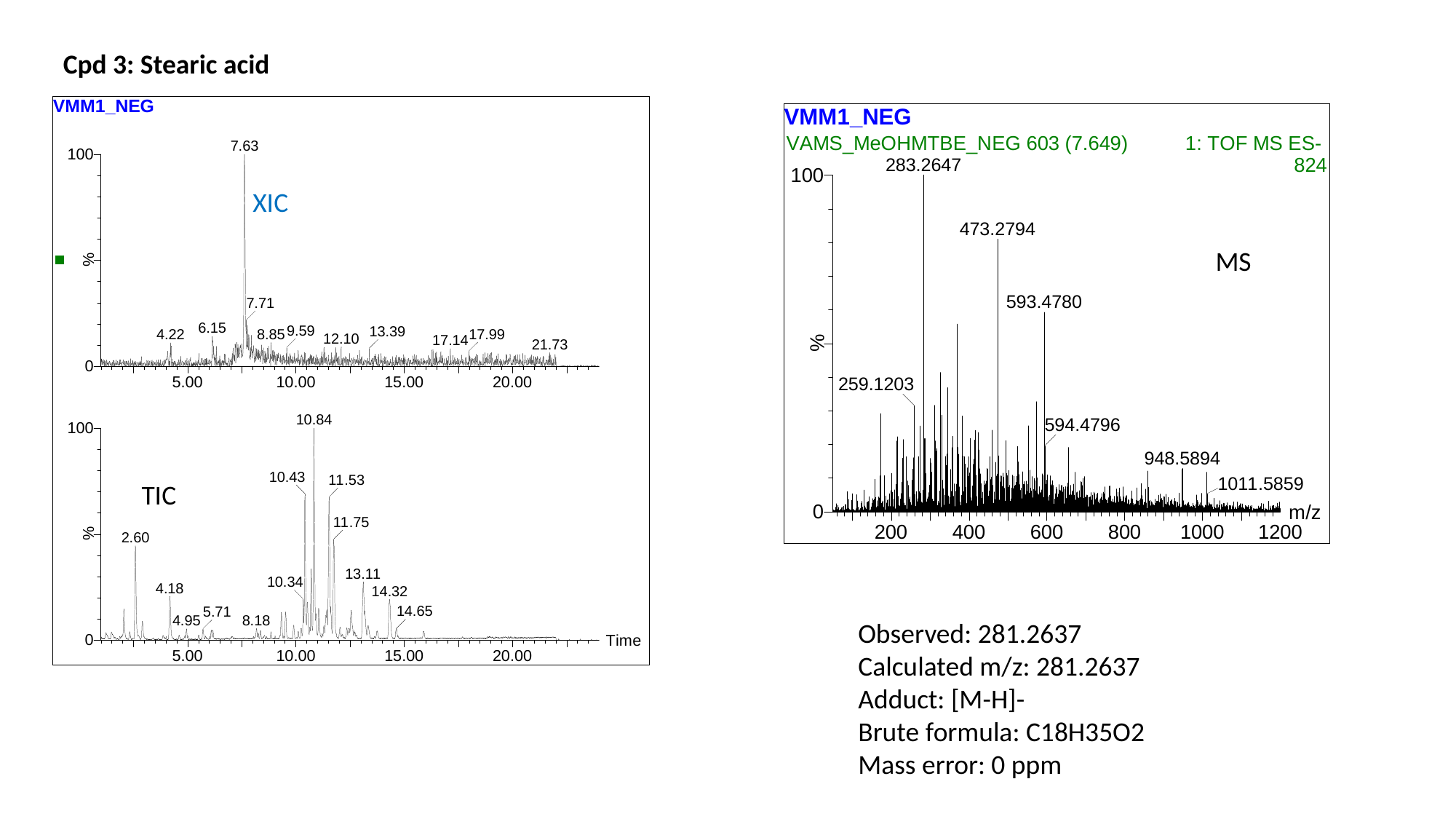

Cpd 3: Stearic acid
XIC
MS
TIC
Observed: 281.2637
Calculated m/z: 281.2637
Adduct: [M-H]-
Brute formula: C18H35O2
Mass error: 0 ppm

## Slide 7
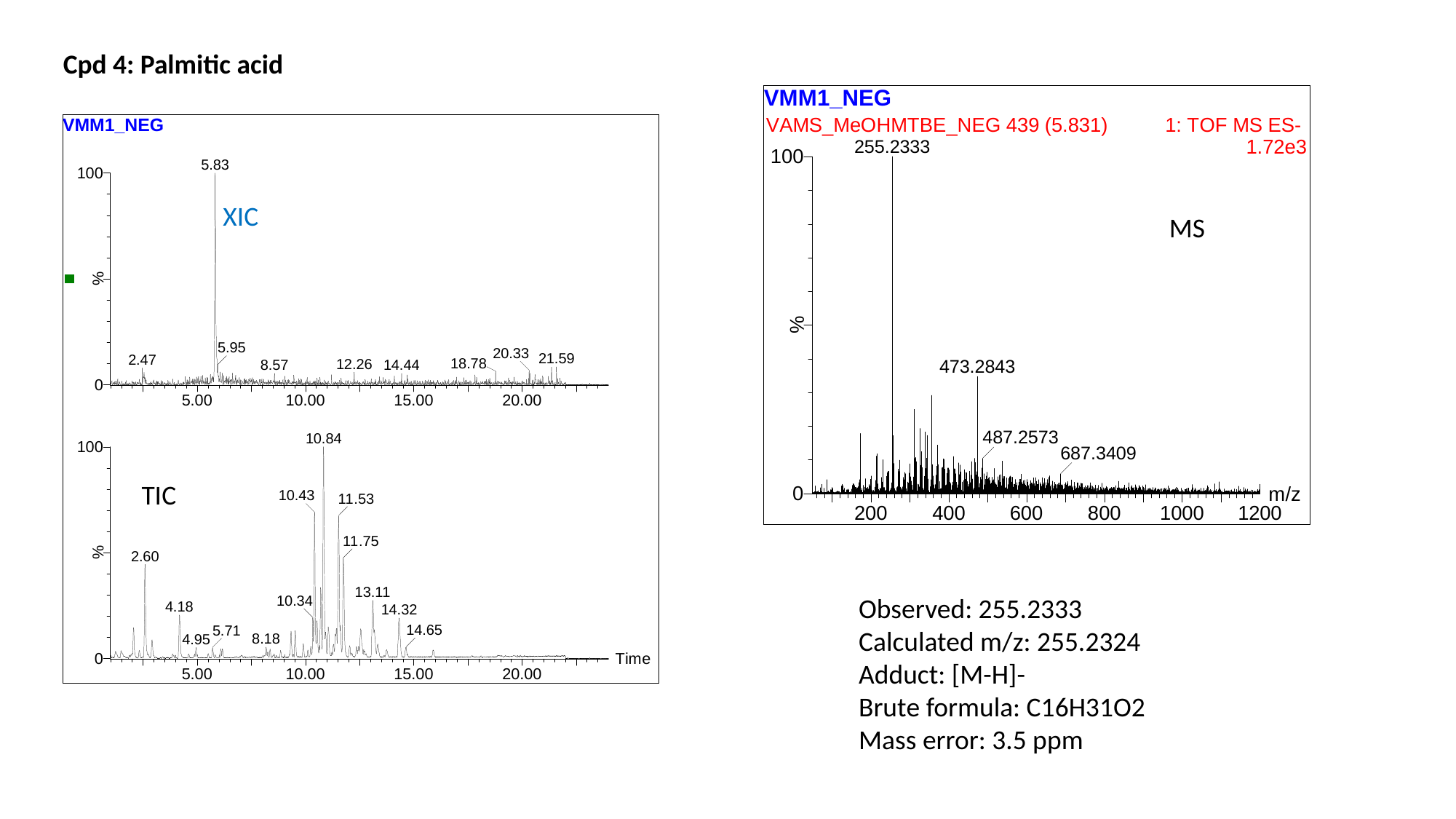

Cpd 4: Palmitic acid
XIC
MS
TIC
Observed: 255.2333
Calculated m/z: 255.2324
Adduct: [M-H]-
Brute formula: C16H31O2
Mass error: 3.5 ppm

## Slide 8
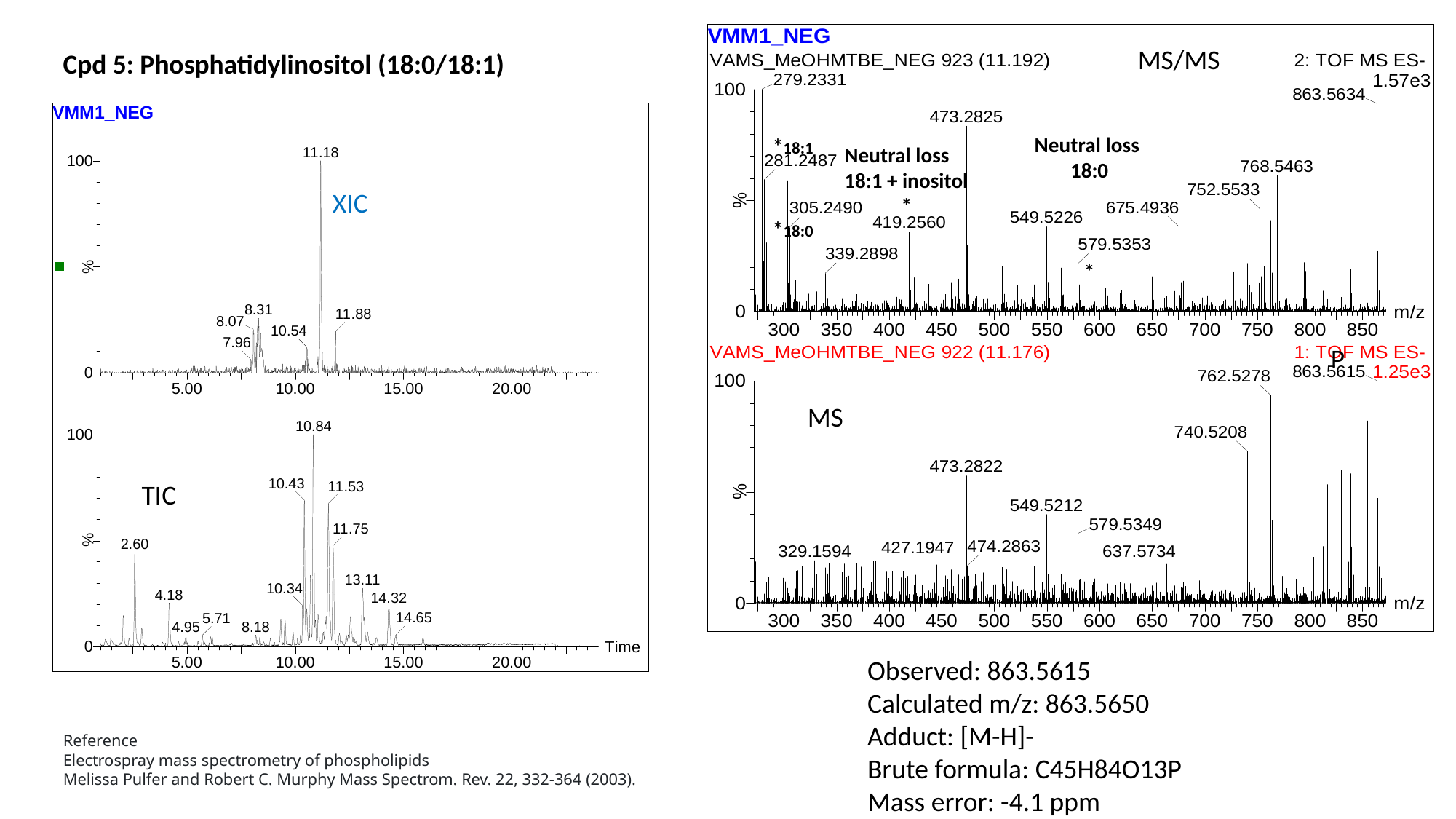

MS/MS
Cpd 5: Phosphatidylinositol (18:0/18:1)
Neutral loss
18:0
*
*18:1
Neutral loss
18:1 + inositol
*
XIC
*18:0
P
MS
TIC
Observed: 863.5615
Calculated m/z: 863.5650
Adduct: [M-H]-
Brute formula: C45H84O13P
Mass error: -4.1 ppm
Reference
Electrospray mass spectrometry of phospholipids
Melissa Pulfer and Robert C. Murphy Mass Spectrom. Rev. 22, 332-364 (2003).

## Slide 9
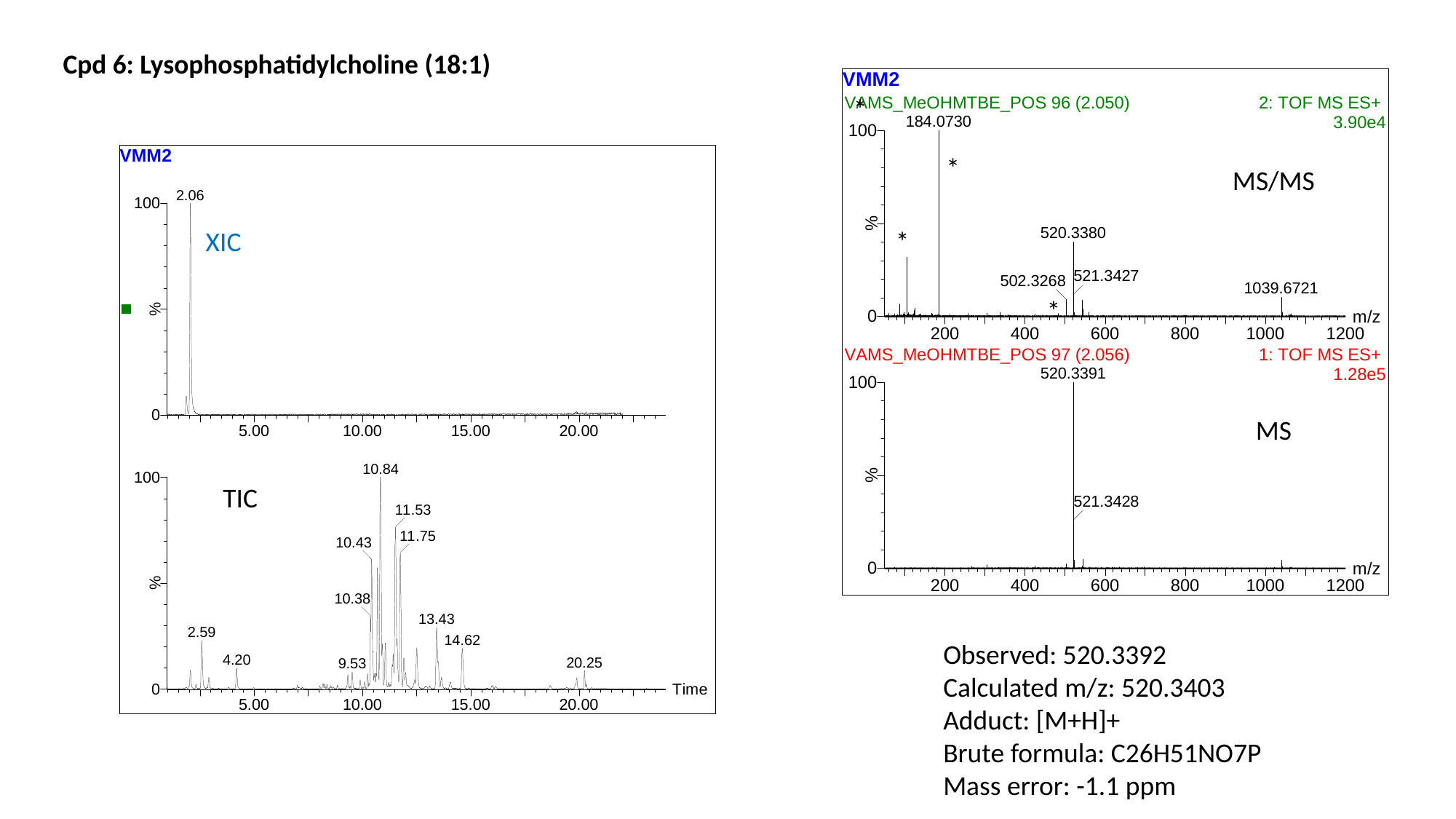

Cpd 6: Lysophosphatidylcholine (18:1)
*
*
MS/MS
XIC
*
*
MS
TIC
Observed: 520.3392
Calculated m/z: 520.3403
Adduct: [M+H]+
Brute formula: C26H51NO7P
Mass error: -1.1 ppm

## Slide 10
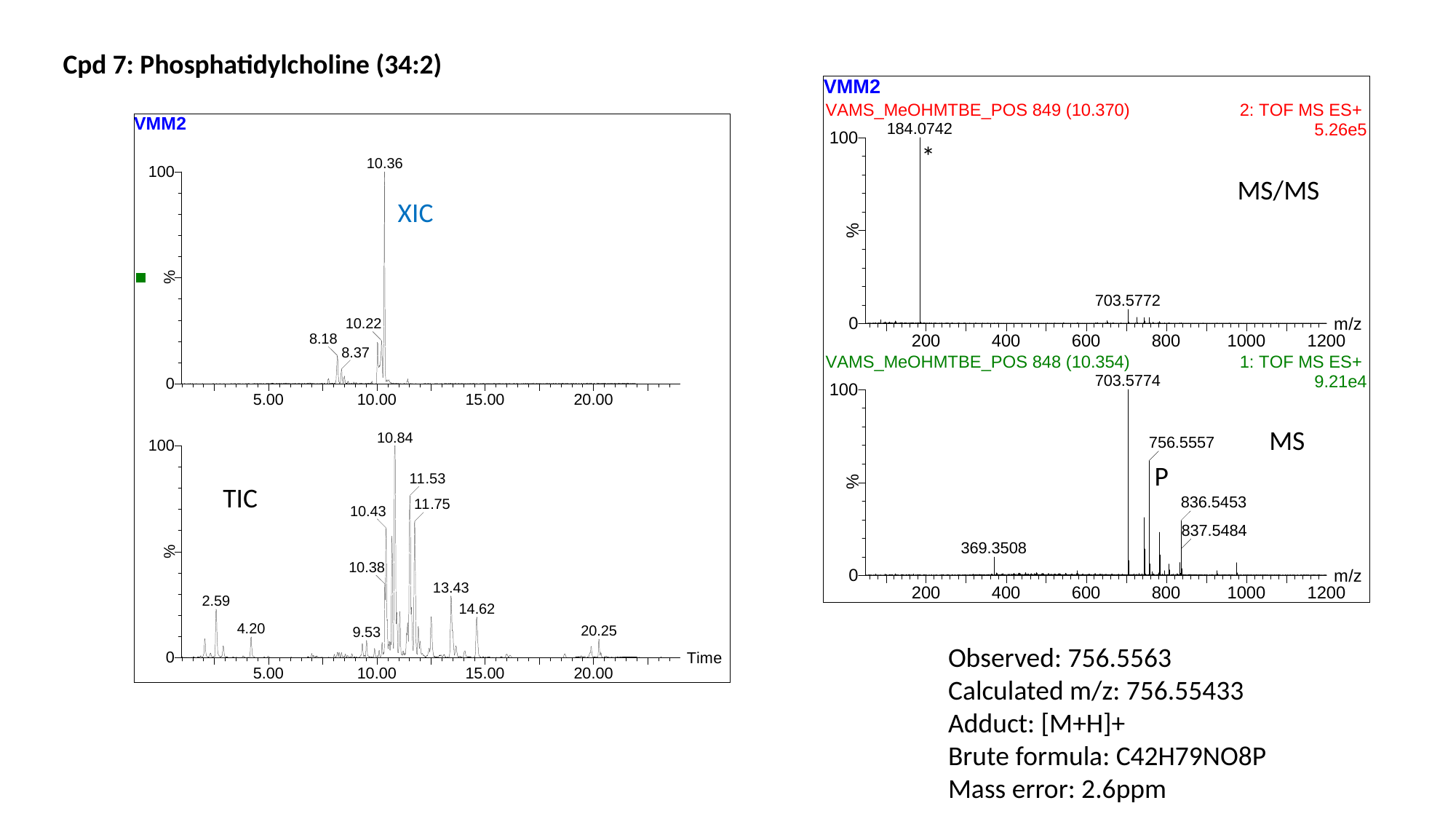

Cpd 7: Phosphatidylcholine (34:2)
*
MS/MS
XIC
MS
P
TIC
Observed: 756.5563
Calculated m/z: 756.55433
Adduct: [M+H]+
Brute formula: C42H79NO8P
Mass error: 2.6ppm

## Slide 11
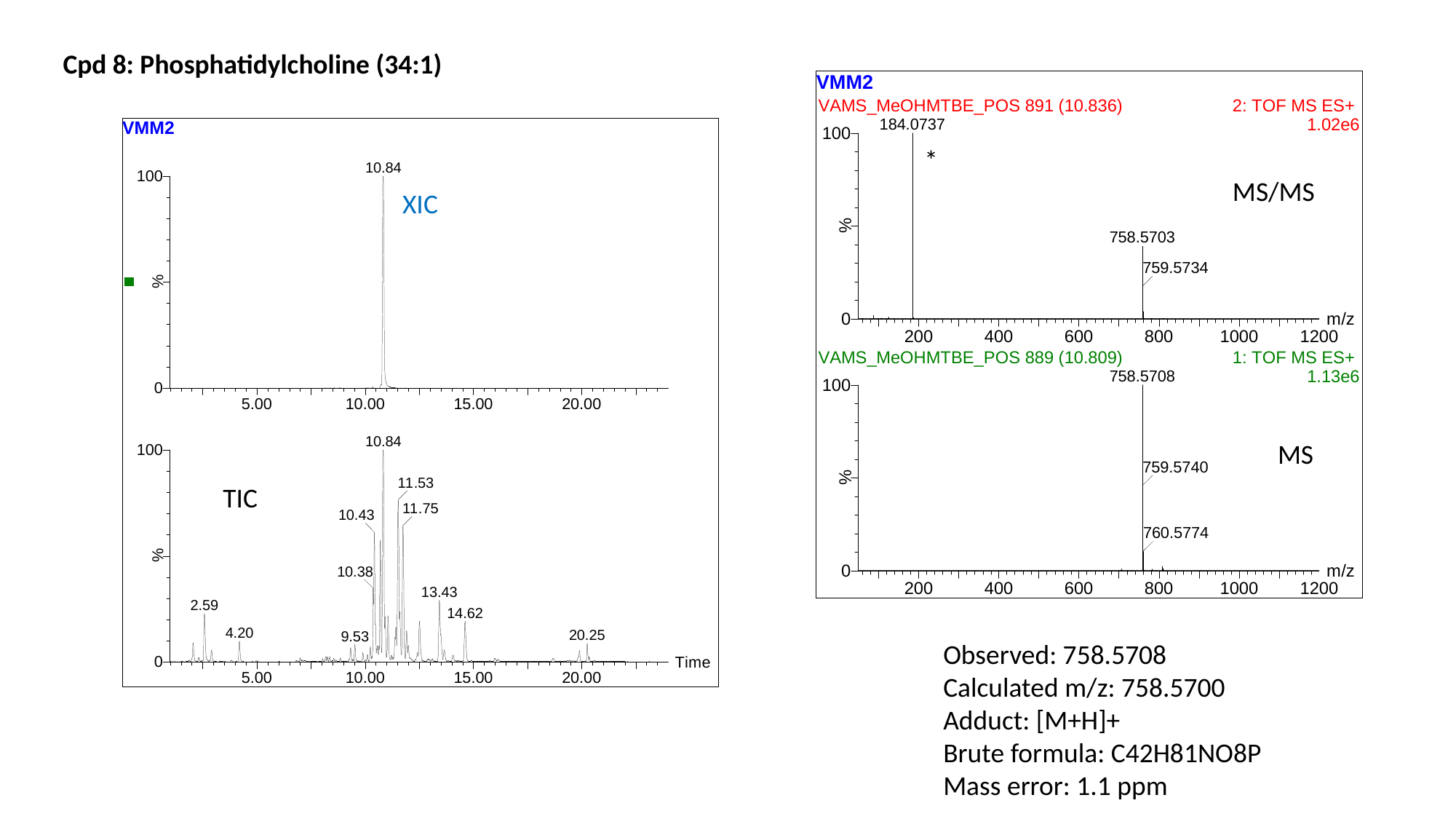

Cpd 8: Phosphatidylcholine (34:1)
*
MS/MS
XIC
MS
TIC
Observed: 758.5708
Calculated m/z: 758.5700
Adduct: [M+H]+
Brute formula: C42H81NO8P
Mass error: 1.1 ppm

## Slide 12
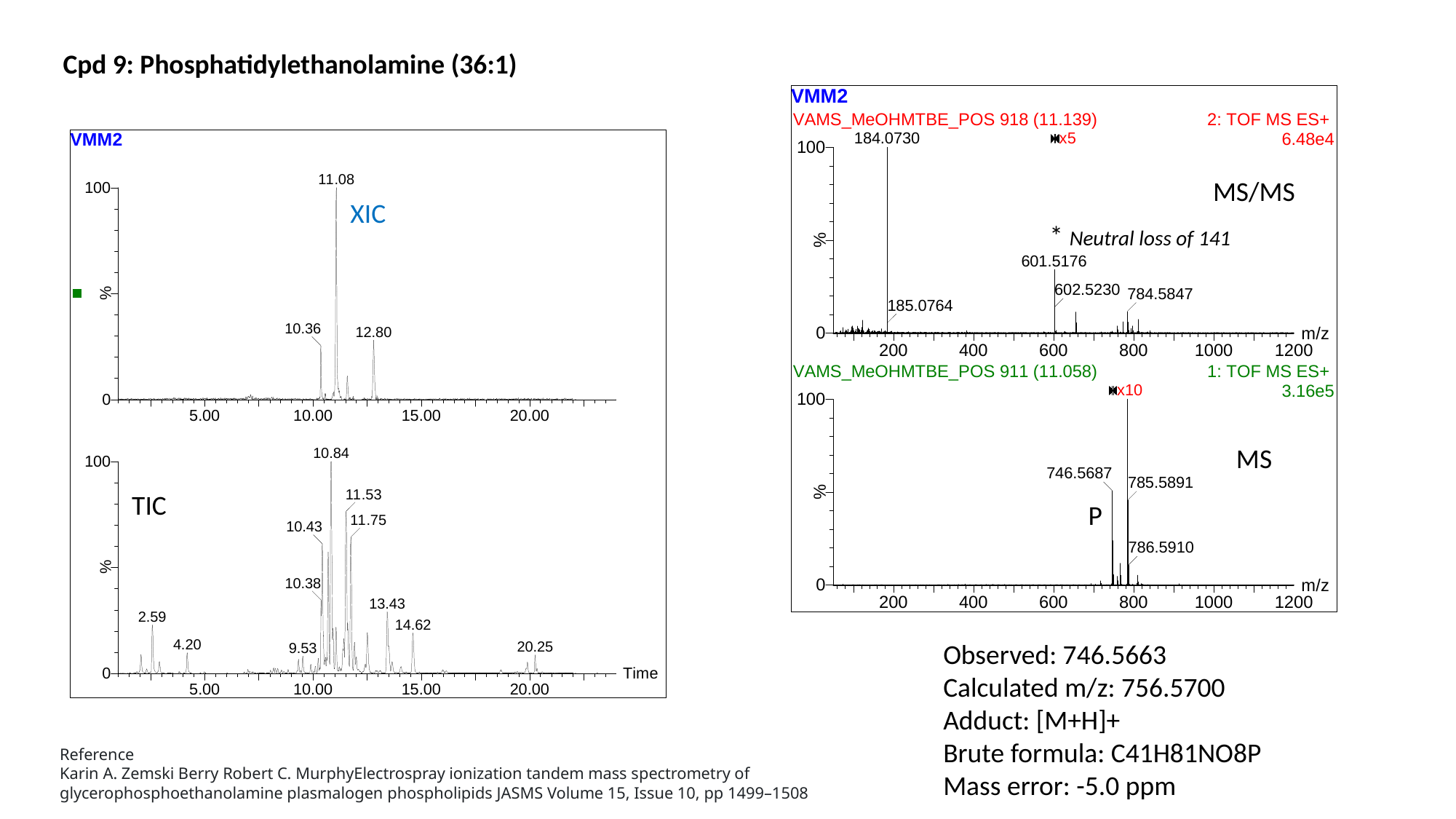

Cpd 9: Phosphatidylethanolamine (36:1)
MS/MS
XIC
* Neutral loss of 141
MS
TIC
P
Observed: 746.5663
Calculated m/z: 756.5700
Adduct: [M+H]+
Brute formula: C41H81NO8P
Mass error: -5.0 ppm
Reference
Karin A. Zemski Berry Robert C. MurphyElectrospray ionization tandem mass spectrometry of glycerophosphoethanolamine plasmalogen phospholipids JASMS Volume 15, Issue 10, pp 1499–1508

## Slide 13
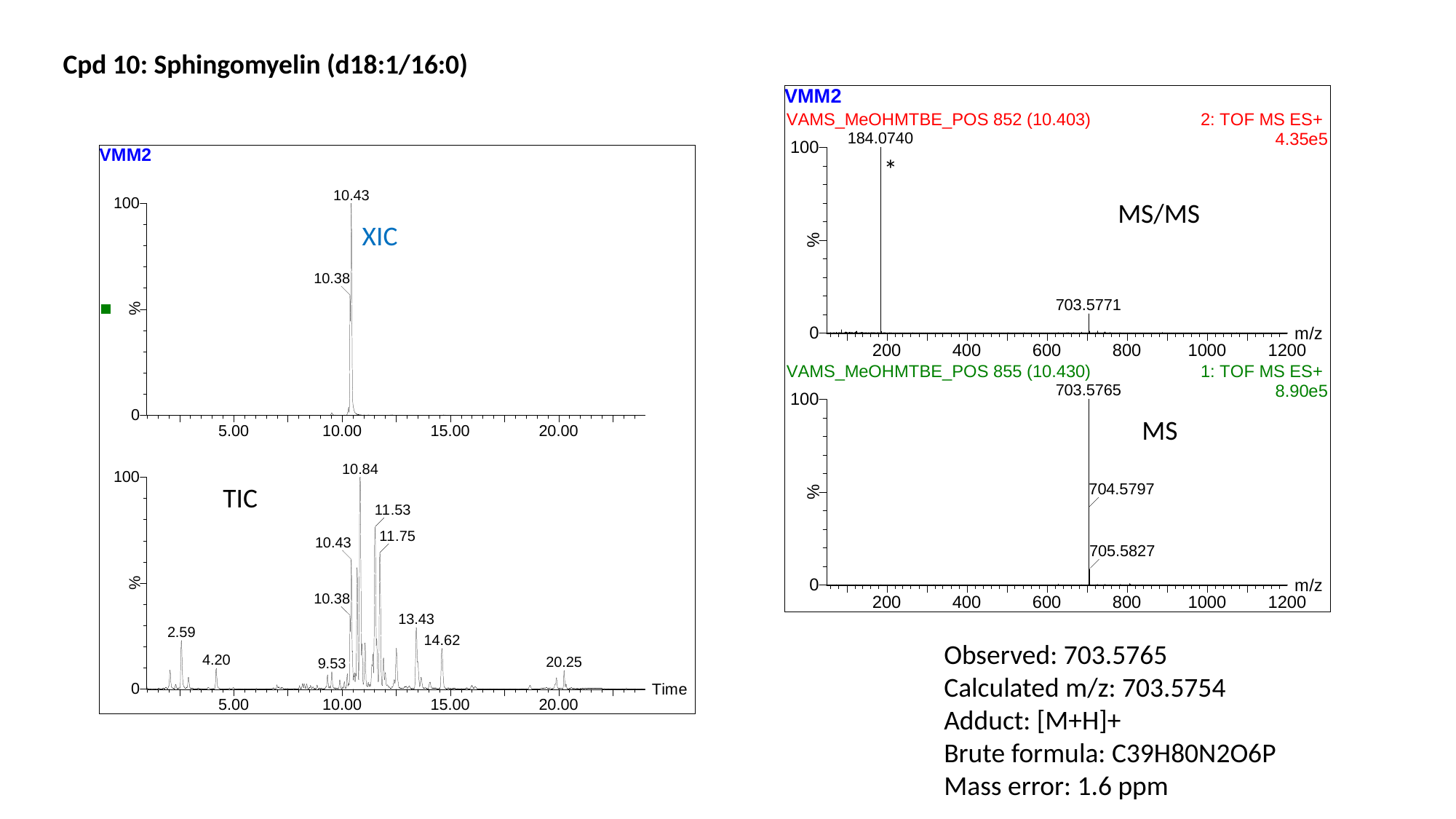

Cpd 10: Sphingomyelin (d18:1/16:0)
*
MS/MS
XIC
MS
TIC
Observed: 703.5765
Calculated m/z: 703.5754
Adduct: [M+H]+
Brute formula: C39H80N2O6P
Mass error: 1.6 ppm

## Slide 14
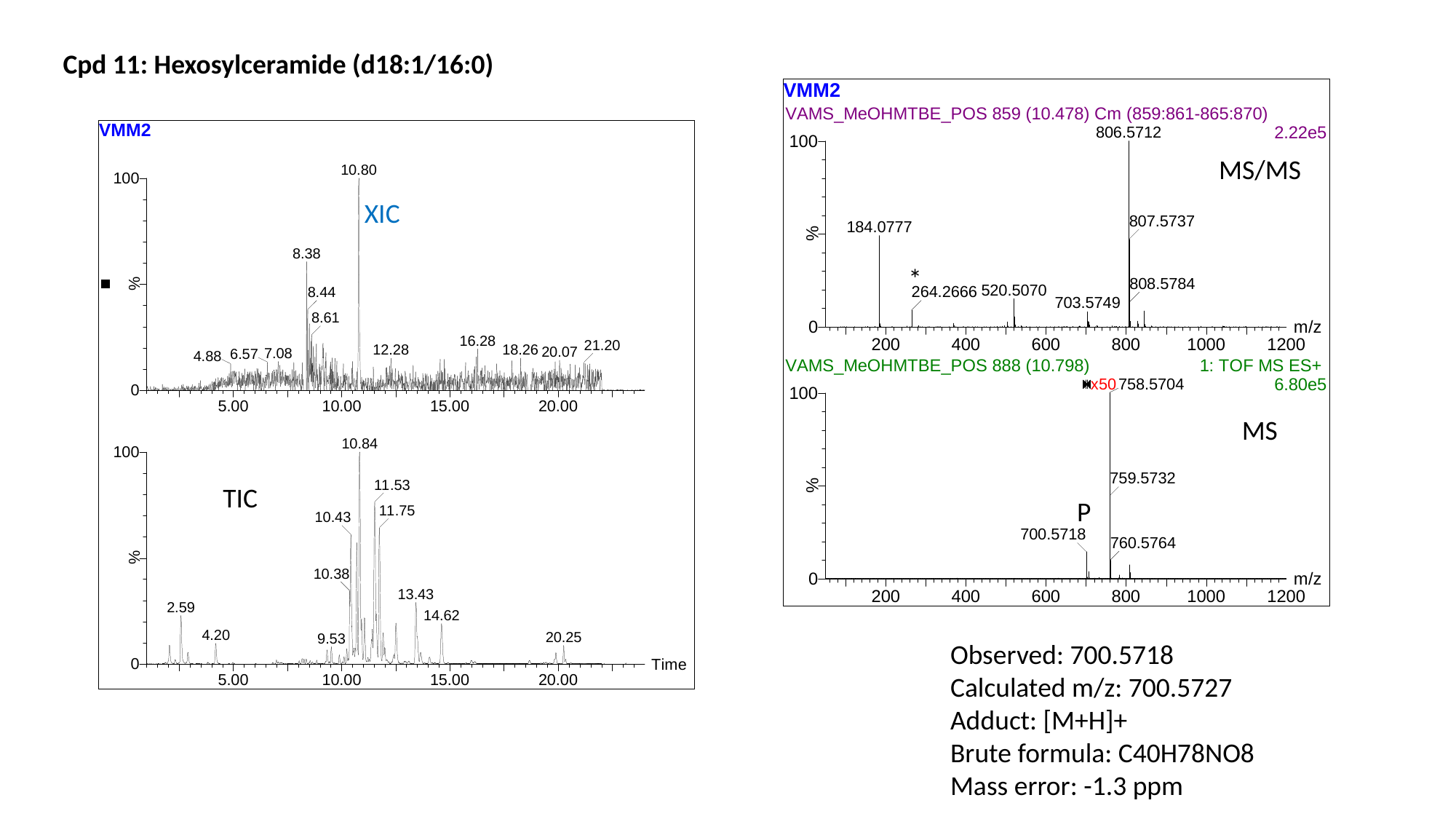

Cpd 11: Hexosylceramide (d18:1/16:0)
MS/MS
XIC
*
MS
TIC
P
Observed: 700.5718
Calculated m/z: 700.5727
Adduct: [M+H]+
Brute formula: C40H78NO8
Mass error: -1.3 ppm

## Slide 15
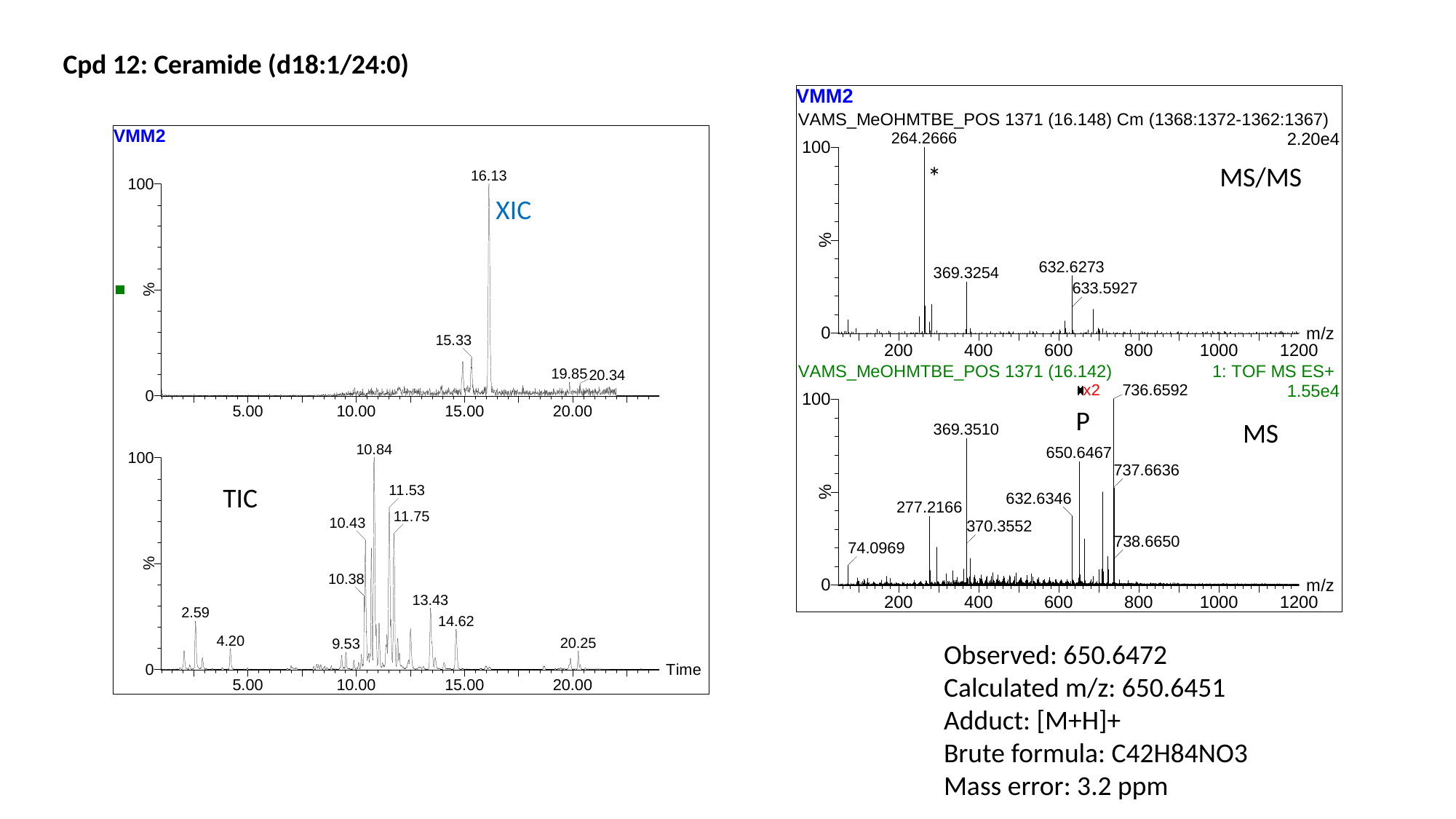

Cpd 12: Ceramide (d18:1/24:0)
*
MS/MS
XIC
P
MS
TIC
Observed: 650.6472
Calculated m/z: 650.6451
Adduct: [M+H]+
Brute formula: C42H84NO3
Mass error: 3.2 ppm

## Slide 16
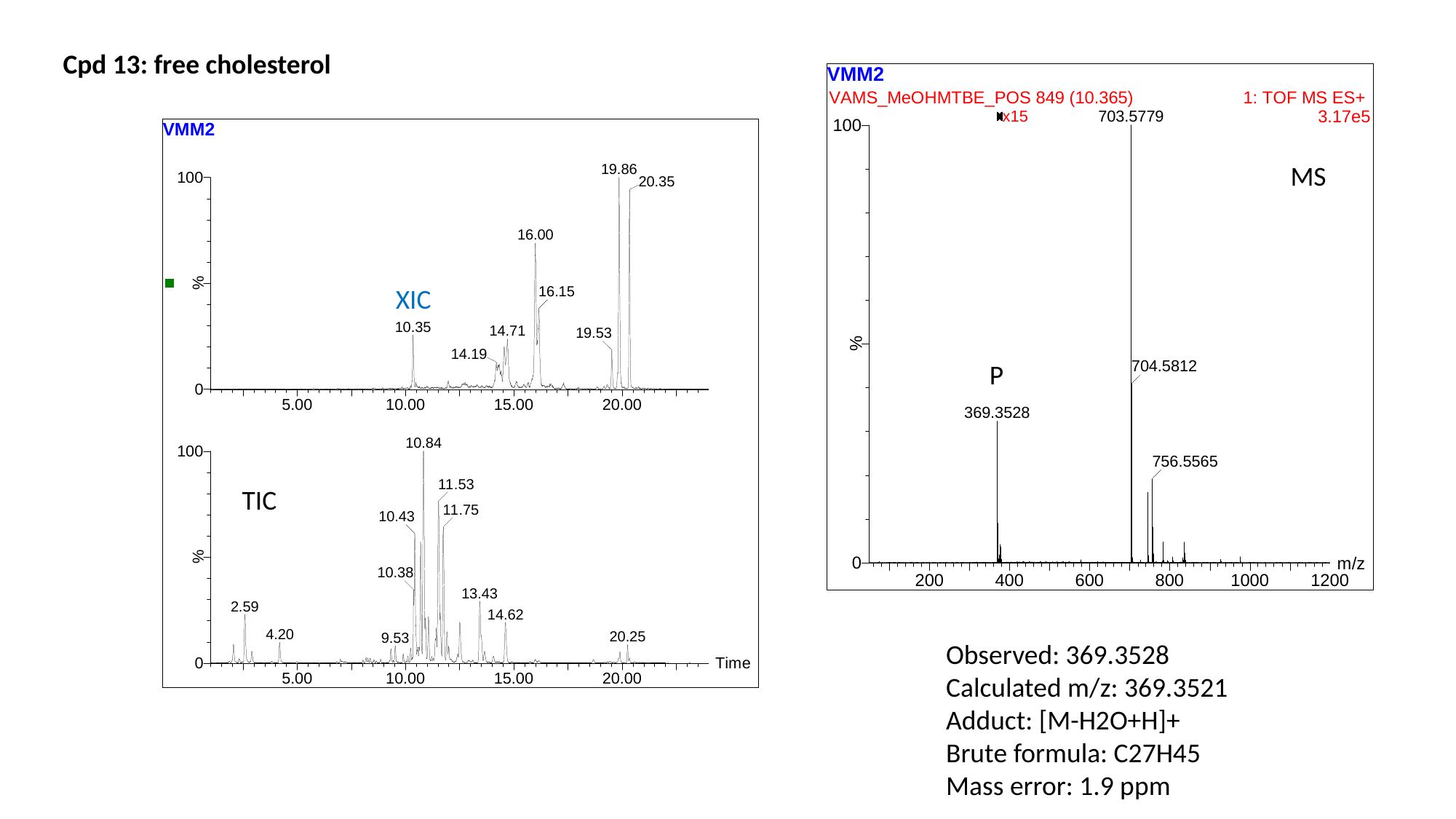

Cpd 13: free cholesterol
MS
XIC
P
TIC
Observed: 369.3528
Calculated m/z: 369.3521
Adduct: [M-H2O+H]+
Brute formula: C27H45
Mass error: 1.9 ppm

## Slide 17
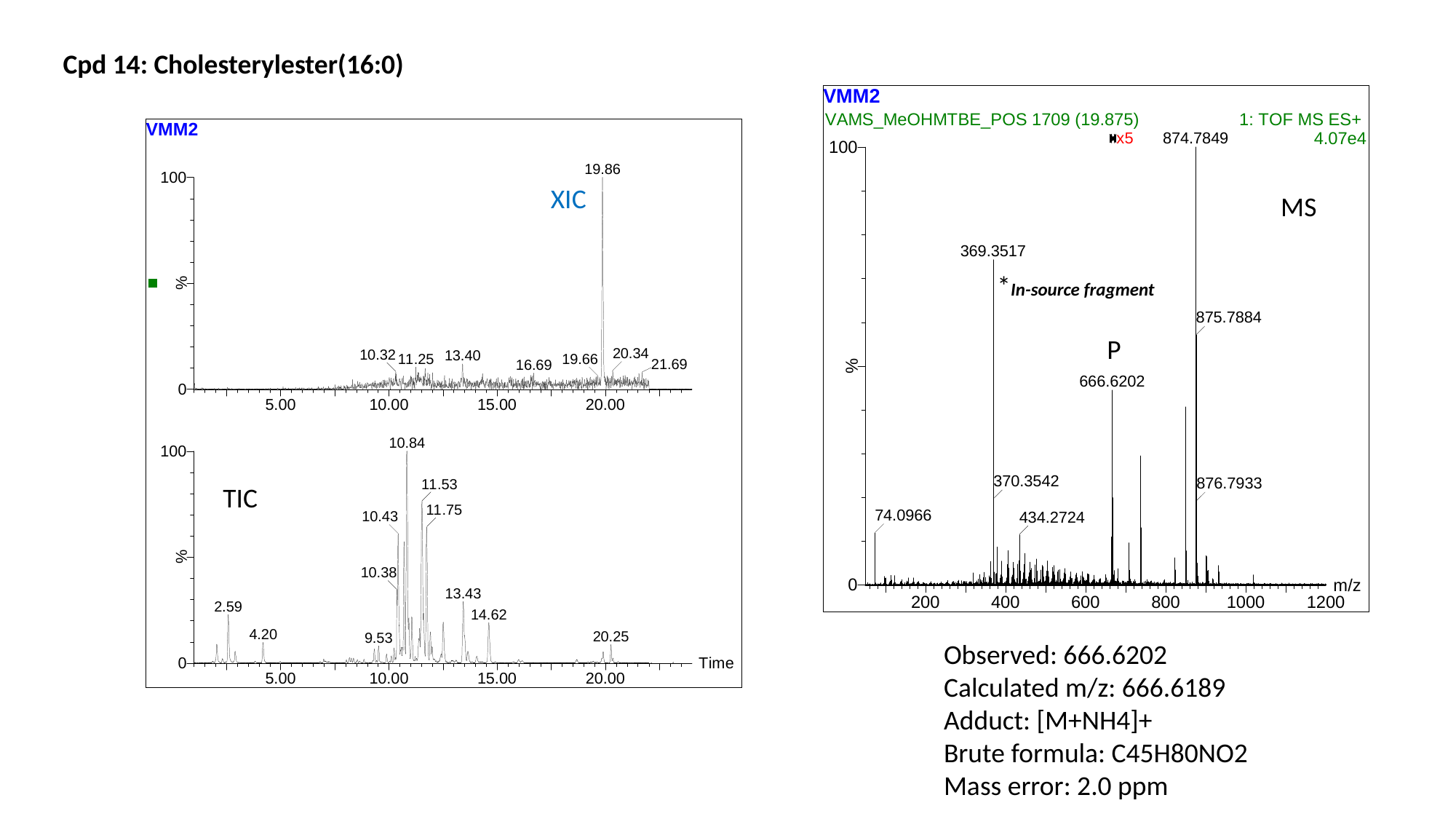

Cpd 14: Cholesterylester(16:0)
XIC
MS
*In-source fragment
P
TIC
Observed: 666.6202
Calculated m/z: 666.6189
Adduct: [M+NH4]+
Brute formula: C45H80NO2
Mass error: 2.0 ppm

## Slide 18
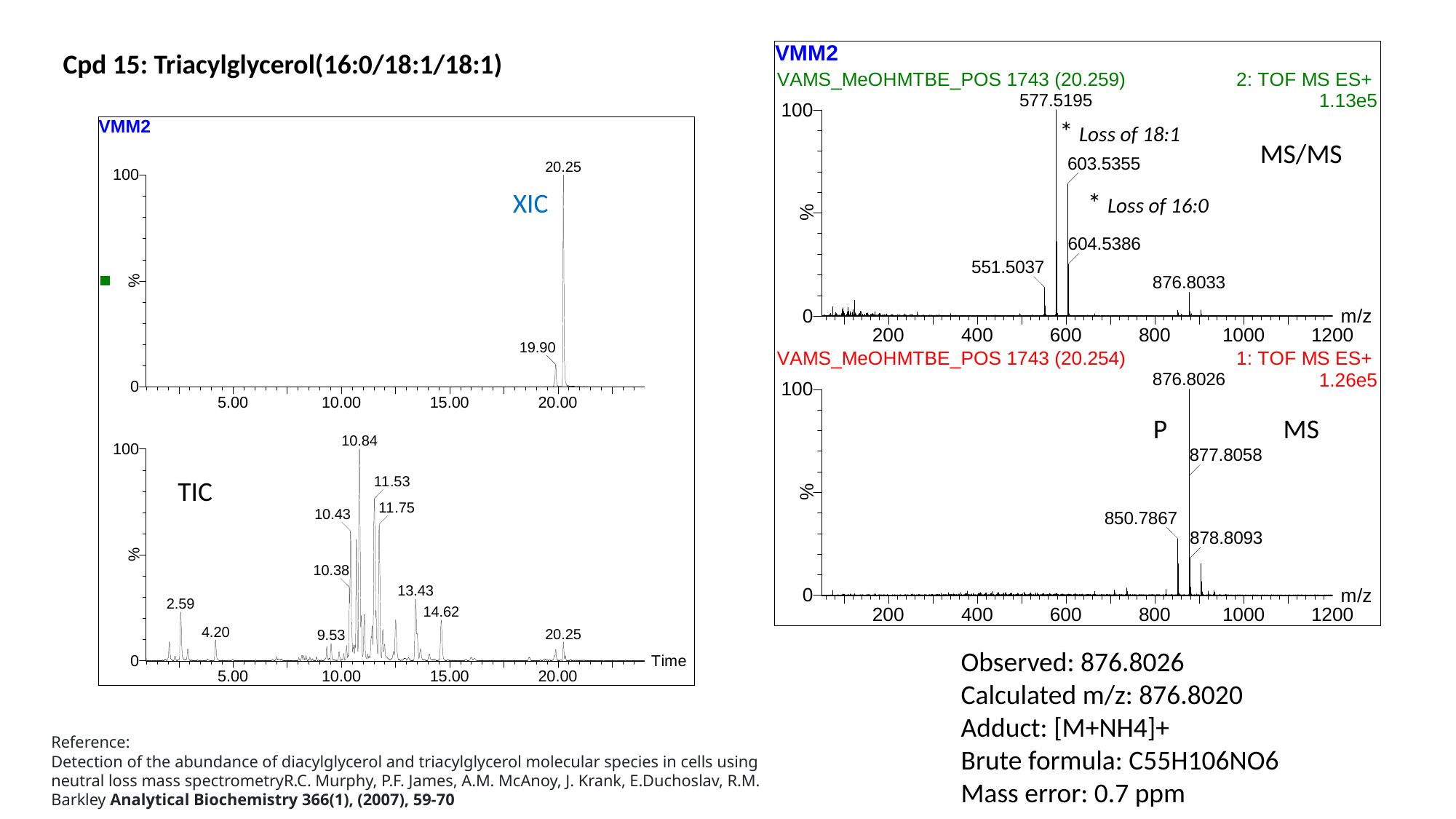

Cpd 15: Triacylglycerol(16:0/18:1/18:1)
* Loss of 18:1
MS/MS
XIC
* Loss of 16:0
P
MS
TIC
Observed: 876.8026
Calculated m/z: 876.8020
Adduct: [M+NH4]+
Brute formula: C55H106NO6
Mass error: 0.7 ppm
Reference:
Detection of the abundance of diacylglycerol and triacylglycerol molecular species in cells using neutral loss mass spectrometryR.C. Murphy, P.F. James, A.M. McAnoy, J. Krank, E.Duchoslav, R.M. Barkley Analytical Biochemistry 366(1), (2007), 59-70

## Slide 19
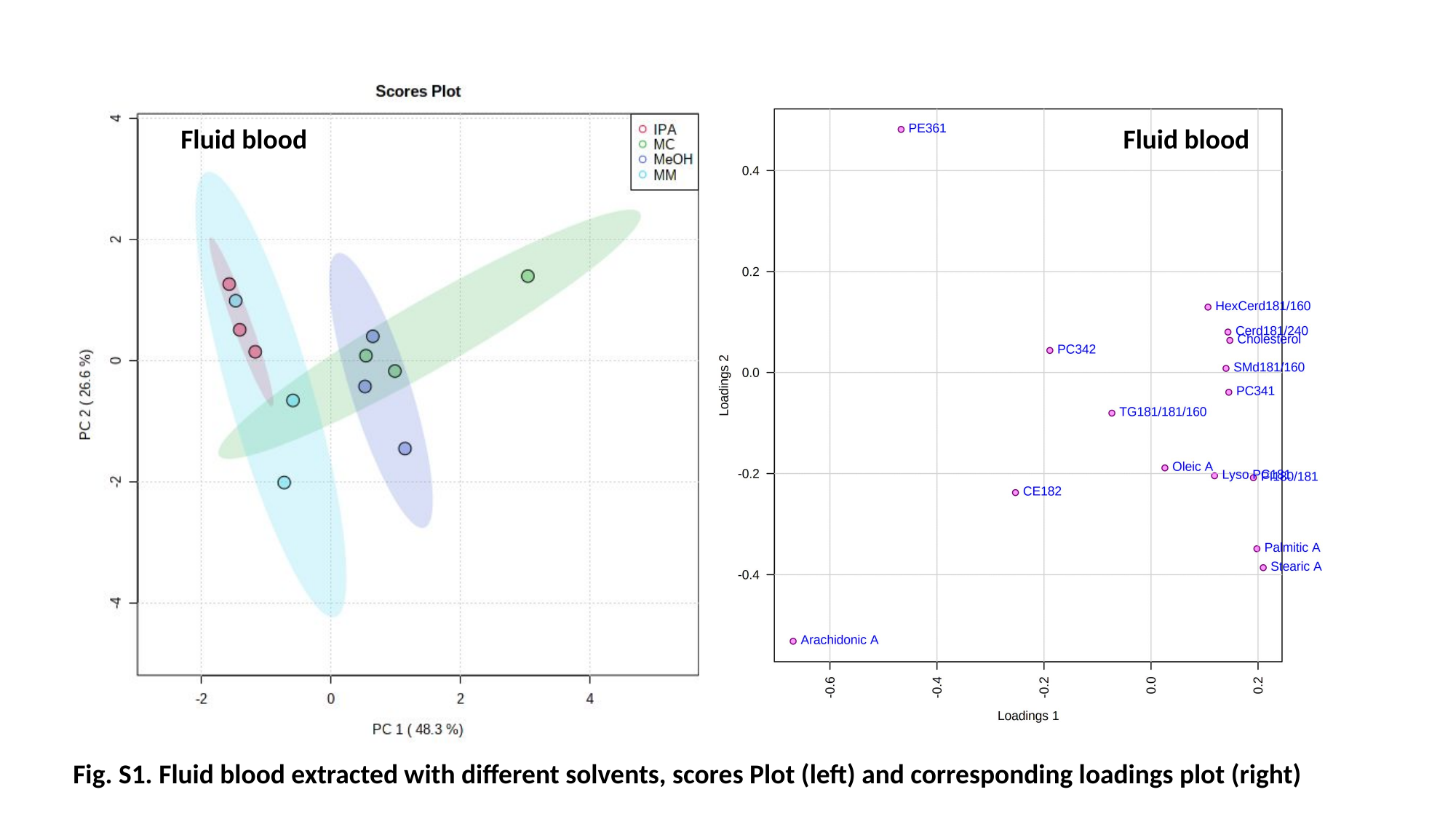

Fluid blood
Fluid blood
Fig. S1. Fluid blood extracted with different solvents, scores Plot (left) and corresponding loadings plot (right)

## Slide 20
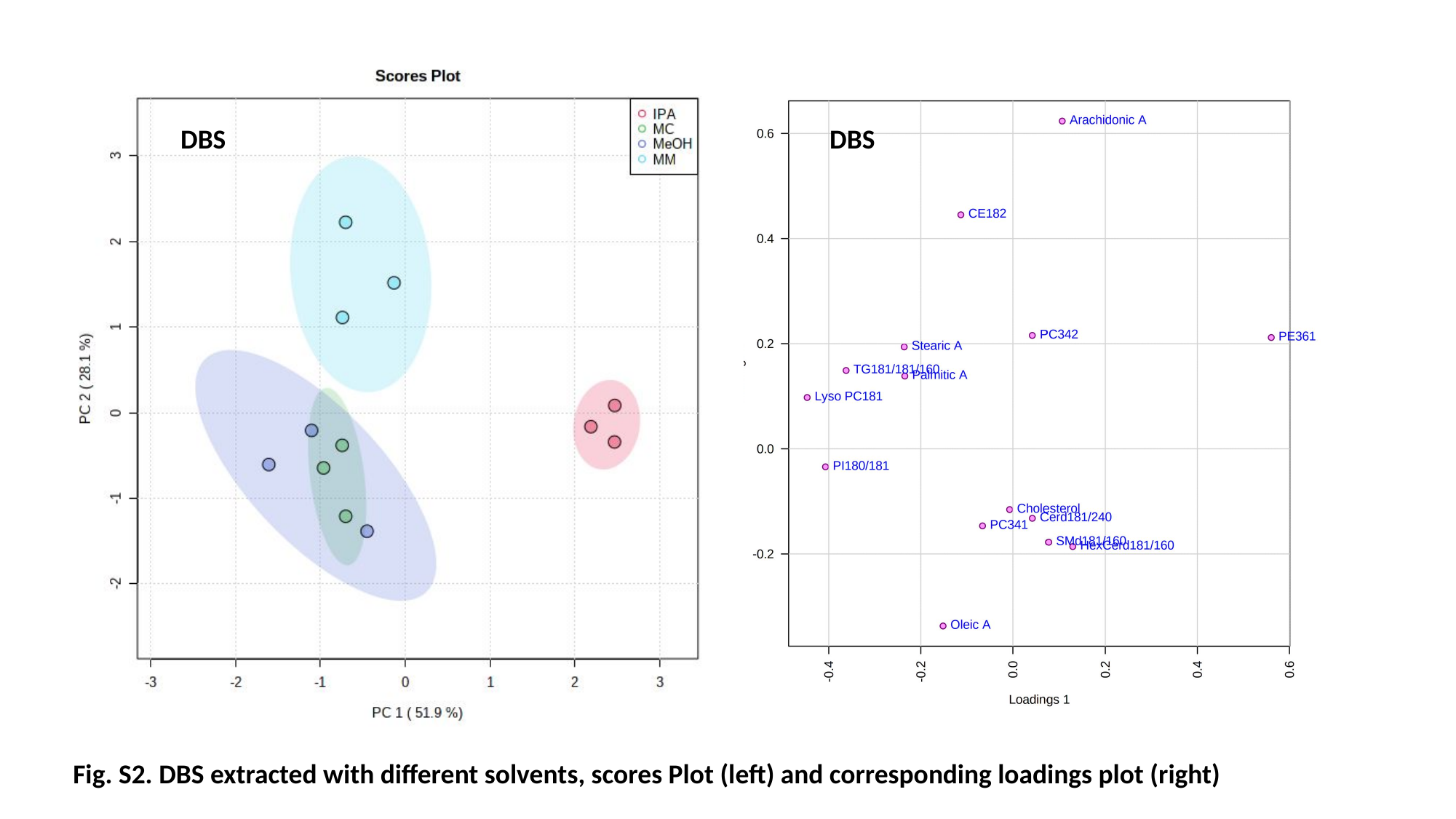

DBS
DBS
DBS
Fig. S2. DBS extracted with different solvents, scores Plot (left) and corresponding loadings plot (right)

## Slide 21
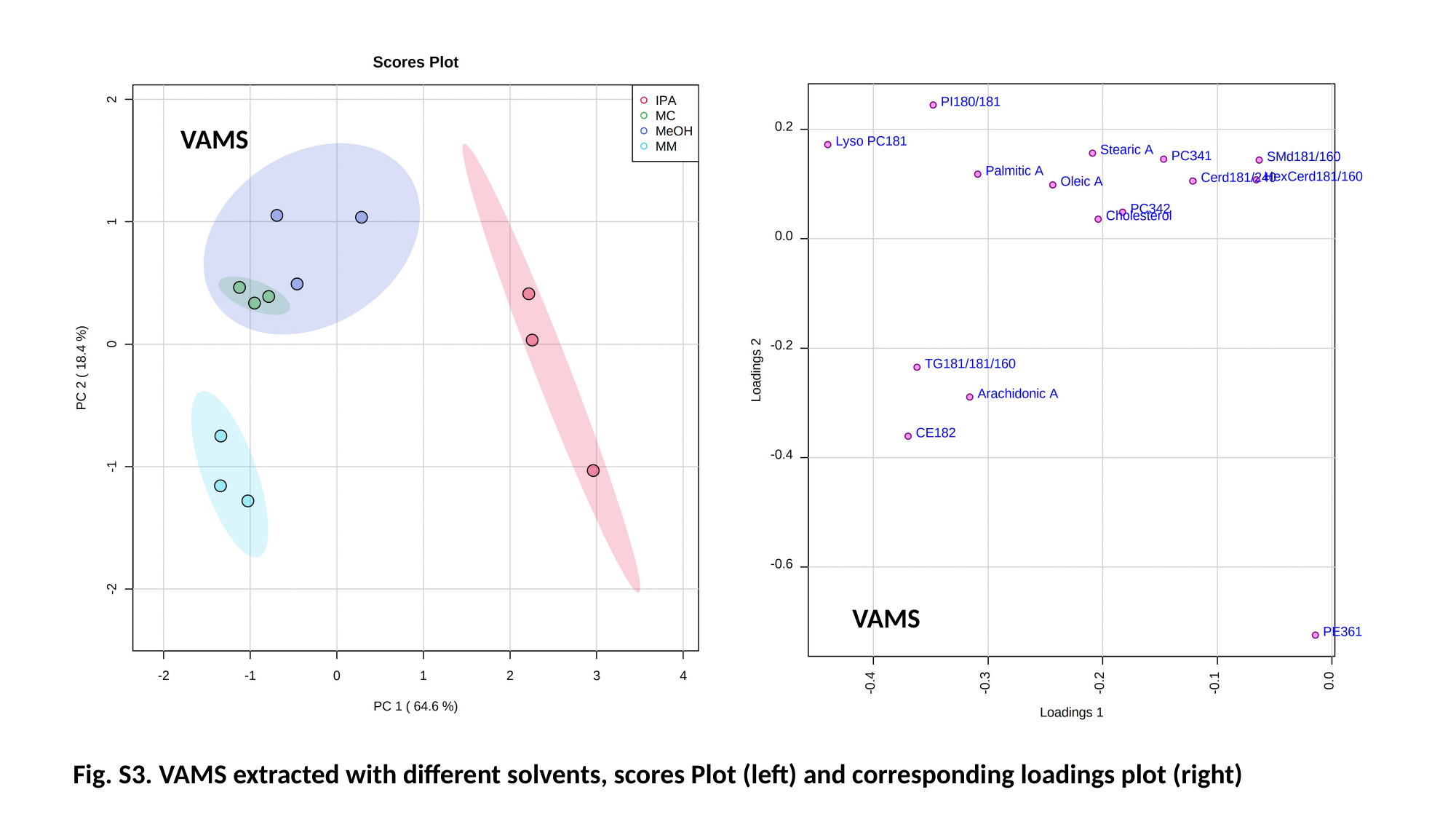

VAMS
VAMS
Fig. S3. VAMS extracted with different solvents, scores Plot (left) and corresponding loadings plot (right)
